# Ethical Considerations in Infodemic Management: Systematic Scoping Review

**DOI:** 10.2196/56307

**Published:** 2024-08-29

**Authors:** Federico Germani, Giovanni Spitale, Sandra Varaidzo Machiri, Calvin Wai Loon Ho, Isabella Ballalai, Nikola Biller-Andorno, Andreas Alois Reis

**Affiliations:** 1 Institute of Biomedical Ethics and History of Medicine, University of Zurich Zurich Switzerland; 2 Unit for High Impact Events Preparedness, Department of Epidemic and Pandemic Preparedness and Prevention, World Health Organization Genève Switzerland; 3 Monash Law School, Monash University Melbourne Australia; 4 Brazilian Immunization Society Sao Paolo Brazil; 5 Health Ethics and Governance Unit Department of Research for Health World Health Organization Genève Switzerland

**Keywords:** World Health Organization, ethics, infodemic management, social listening, review, infodemic, health emergency, health emergencies, misinformation, disinformation, scoping review, ethical principles, community engagement, empowerment, data privacy, effectiveness

## Abstract

**Background:**

During health emergencies, effective infodemic management has become a paramount challenge. A new era marked by a rapidly changing information ecosystem, combined with the widespread dissemination of misinformation and disinformation, has magnified the complexity of the issue. For infodemic management measures to be effective, acceptable, and trustworthy, a robust framework of ethical considerations is needed.

**Objective:**

This systematic scoping review aims to identify and analyze ethical considerations and procedural principles relevant to infodemic management, ultimately enhancing the effectiveness of these practices and increasing trust in stakeholders performing infodemic management practices with the goal of safeguarding public health.

**Methods:**

The review involved a comprehensive examination of the literature related to ethical considerations in infodemic management from 2002 to 2022, drawing from publications in PubMed, Scopus, and Web of Science. Policy documents and relevant material were included in the search strategy. Papers were screened against inclusion and exclusion criteria, and core thematic areas were systematically identified and categorized following PRISMA (Preferred Reporting Items for Systematic Reviews and Meta-Analyses) guidelines. We analyzed the literature to identify *substantive ethical principles* that were crucial for guiding actions in the realms of infodemic management and social listening, as well as related *procedural ethical principles*. In this review, we consider ethical principles that are extensively deliberated upon in the literature, such as equity, justice, or respect for autonomy. However, we acknowledge the existence and relevance of procedural practices, which we also consider as ethical principles or practices that, when implemented, enhance the efficacy of infodemic management while ensuring the respect of substantive ethical principles.

**Results:**

Drawing from 103 publications, the review yielded several key findings related to ethical principles, approaches, and guidelines for practice in the context of infodemic management. Community engagement, empowerment through education, and inclusivity emerged as procedural principles and practices that enhance the quality and effectiveness of communication and social listening efforts, fostering trust, a key emerging theme and crucial ethical principle. The review also emphasized the significance of transparency, privacy, and cybersecurity in data collection.

**Conclusions:**

This review underscores the pivotal role of ethics in bolstering the efficacy of infodemic management. From the analyzed body of literature, it becomes evident that ethical considerations serve as essential instruments for cultivating trust and credibility while also facilitating the medium-term and long-term viability of infodemic management approaches.

## Introduction

### Background

In an age dominated by the digital dissemination of information and in an information ecosystem [1] in which the digital divide continues to be a global challenge, a new term has recently emerged, one that reflects the profound impact of the digital age on our information landscape: “infodemics,” derived from the fusion of the terms *information* and *epidemic* [2,3]. The World Health Organization (WHO) defines an infodemic as the surge of information, both accurate and false, that inundates the public during acute health events such as outbreaks and epidemics. These infodemics hold far-reaching consequences, affecting public health, shaping societal decision-making, and influencing individual behaviors [4].

Infodemics can have a detrimental effect on public health efforts by raising questions, concerns, and doubts, which, if unresolved, may lead to information voids, alongside an overabundance of information, accurate or not, that can incite panic and confusion while hindering the dissemination of vital information [5-7]. In the digital era, distinguishing fact from fiction is a difficult task for the public [8,9], and the successful recognition of the accuracy of information requires information literacy and critical thinking [10-12]. Equally complex is the role of public health institutions and infodemic managers, as they navigate a polarized society that often rejects well-intentioned, safety-focused information [13].

Infodemic management refers to the processes and strategies put in place to monitor and improve the information ecosystem, including handling and controlling the spread of misinformation and excessive information during health crises [14]. Social listening involves monitoring and analyzing online and offline conversations (eg, on social media) to gain insights into public sentiment, concerns, and behaviors during such crises [15]. Social listening is an integral component of infodemic management, as it is an important source of insights to improve public health interventions [15,16].

The importance of ethics in infodemic management has been underscored amid the COVID-19 pandemic [17-19], as the lack of the integration of ethical approaches in the management of infodemics has diminished the potential for long-term effectiveness of these strategies, leading to a decline in trust for those same institutions safeguarding public health [20].

Ethical considerations encompass a broad scope, including determining when and under what circumstances intervention in the public information space is justifiable. Privacy, autonomy, trust, and the potential for censorship are all salient issues [21-24]. Neglecting these ethical dimensions can have profound consequences, eroding public trust and inadvertently causing harm [25]. Therefore, it is crucial to explore how ethical awareness may contribute to improving the effectiveness of these practices and how ethics can offer practical tools to solve the problems posed by infodemics. Infodemic management is a discipline driven by a moral imperative to improve the quality of the information ecosystem, thereby ensuring better public health outcomes and saving lives. In this review, ethical considerations are therefore defined as reasonings on morally significant principles intended to shape and guide the actions of stakeholders involved in and executing infodemic management practices [26]. We consider as “ethical considerations” not only those concerning the implementation of the infodemic management moral imperative but also those that enhance the short-term and long-term effectiveness of infodemic management.

The concept of infodemics gained prominence during the COVID-19 pandemic [2,7,14] and is expected to remain a pressing concern even as COVID-19 is no longer a major public health emergency [7,27,28]. Given the recent focus on integrating ethics into infodemic management and social listening [25,29], coupled with the expanding scope of infodemic-related challenges and the widespread adoption of social listening techniques for monitoring public health concerns and behaviors, it is imperative to investigate how existing literature addresses the ethical dimensions of infodemic management and social listening. This exploration can provide valuable insights to guide the integration of ethics into infodemic management and social listening practices while ensuring their effectiveness. The aim of this systematic scoping review is to pinpoint ethical considerations that have proven beneficial in the past, thereby informing and guiding future advancements in the field.

Recognizing the global urgency of integrating ethics into infodemic management and social listening practices, the WHO established the expert group (EG) on ethical considerations in infodemic management and social listening in 2023 [29]. This EG, coordinated by the Unit for High Impact Events Preparedness and the Health Ethics and Governance Unit at WHO, highlights the need for a comprehensive and ethically sound response to infodemics, with an eye to improve infodemic management practices as devised during the acute phases of the COVID-19 pandemic. The EG is developing practical tools to guide infodemic managers to ethically monitor infodemic trends and guide interventions. The EG discusses emerging dilemmas, practical applications of guidance in the field, and areas requiring deeper exploration. Importantly, the EG is working toward the goal of creating WHO ethics guidance and a practical implementation framework, along with accompanying tools, for infodemic managers and public health institutions involved in shaping or conducting infodemic management. The systematic scoping review described in this paper is essential for the work of the WHO’s EG on ethical considerations in infodemic management and social listening by providing literature-driven insights and grounding their discussions and guidance in empirical evidence. In addition to detailing the practical initiatives already implemented to integrate ethics into the practice of infodemic management and social listening, this systematic scoping review also aims to describe the global health research community’s perspective and understanding of the ethical dimensions crucial to infodemic management, thus blending practical, theoretical, and experimental perspectives to advance the field.

### Objectives

The primary objective of this review is to identify, categorize, and analyze the ethical challenges and issues related to infodemics and their management. Our scope primarily covers literature published between 2002 and 2022, extracted from PubMed, Web of Science, and Scopus and enriched by a substantial amount of gray literature and policy documents contributed by distinguished experts in the field.

## Methods

### A Methodological Note on Our Approach to Ethics

Traditionally, ethical inquiry in applied ethics has been centered on establishing, defining, and elucidating substantive ethical principles [30,31]. These principles guide ethical decision-making and conduct. Commonly considered substantive principles include equity, justice, beneficence, and respect for autonomy. Within the context of our investigation of the literature concerning ethics and infodemic management, we fundamentally consider these principles, which provide a theoretical foundation for ethical considerations in infodemic management. However, our approach diverges from traditional frameworks by incorporating not only substantive ethical principles but also procedural principles that we consider as “proethical” [32]. While substantive ethical principles offer overarching moral guidance, procedural principles operationalize substantive principles into actionable steps for implementation in practice. In this systematic scoping review, we recognize the significance of both types of ethical considerations in the domains of infodemic management. By systematically examining the literature through the lens of both substantive and procedural principles, we aimed to provide a comprehensive understanding of ethics in the context of infodemic management during emergency health crises. Our analysis goes beyond defining ethics and identifying ethical principles that need to be respected in infodemic management; rather, it seeks to identify practical strategies and methodologies that can enhance the ethical conduct of infodemic management while augmenting the effectiveness of these practices. We used this approach as we acknowledge the dynamic and rapidly evolving nature of ethical challenges in public health and infodemic management specifically.

### Approach

This review intertwines elements from both scoping and systematic reviews. Traditionally, a scoping review maps an expansive and varied body of literature to provide an overview of a broad subject area, identifying knowledge gaps [33]. A systematic review consolidates empirical evidence from a set of studies centered around a precise research question [33]. Our review has the goal of a scoping review and adopts the methodology of a systematic review [34]. We adhered to the PRISMA (Preferred Reporting Items for Systematic Reviews and Meta-Analyses) guidelines [35]. The PRISMA checklist is available in Multimedia Appendix 1 [6,7,16,17,27,28,36-78].

### Query Definition

To initiate the search and extraction process, we used TopicTracker for text mining within PubMed records [79]. Using TopicTracker, we executed our initial query (query v0). All versions of our queries are available in Multimedia Appendix 1. This preliminary attempt harvested 34 papers. Interestingly, only 3 (9%) of these 34 papers were published prior to 2019, focusing on infodemics or social listening aspects that were not tethered to the COVID-19 narrative. A discernible challenge emerged from this exercise: the term “infodemic” was yet to be recognized as a standard keyword to describe research in the field of infodemic management and social listening before 2020. In response, we opted for an expansive approach [80], mining the initial results for synonyms, medical subject heading terms, and lemmas that encapsulate the essence of “infodemic.” Underpinning “infodemic” as the overabundance of information, encompassing misinformation, particularly visible in digital spheres during significant health crises [81], we refined our initial query, leading to the formulation of query v1. This revised query retrieved 151 papers. The earliest papers trace back to 2003, with 54 (36%) of the 151 papers published before the onset of the COVID-19 pandemic. Following a deliberation session with the WHO EG panel on ethical considerations in infodemic management and social listening, we decided to further amplify our search strategy, mining for an expanded array of keywords resonating with “social listening” and incorporating synonymous descriptors for “infodemics,” namely, “information overload,” “information pollution,” “information quality,” “health information,” “information voids,” and “information deficits,” gleaned from a related research project with the goal of mapping infodemic management interventions during health emergencies [82]. The above expansions led to the formulation of query v2. Despite a noticeable expansion in OR keywords, this query fetched only 1 additional paper compared to its predecessor (n=152), suggesting a saturated and robust query formulation. Finally, after another collaborative session with the EG, where query v2 was discussed, we added a few keywords to ensure that the query was not overly restrictive to a few fields of application in the realm of infodemic management and social listening. This iteration, termed query v3, yielded a list of 225 papers. The structure of this query is comprehensively detailed, alongside all previous versions of the query, in Multimedia Appendix 1 and in the study’s protocol stored on our Open Science Framework (OSF) repository [83].

### Query Translation

Once the query was validated by the EG, we translated query v3 for compatibility with Scopus and Web of Science. Detailed representations of these translations are presented in Multimedia Appendix 1 and in the study’s OSF repository [83]. Further enriching our corpus, the EG provided additional material to be added to the list of retrieved items: this comprised not only traditional literature but also gray literature, references from United Nations and WHO work, and recently published work (beyond the cutoff imposed by our study design at the end of 2022). All these elements were integrated into the corpus of the literature retrieved with query v3 and translated queries for Scopus and Web of Science.

### Data Retrieval and Screening of Records

Records extracted from the various sources were collated and stored in a publicly accessible Zotero (Corporation for Digital Scholarship) project [84]. Initial sifting was based on the exclusion criteria applied to the record titles and abstracts. The defined exclusion criteria were as follows: record does not mention social listening or infodemic management (directly or indirectly; see “infodemics (expanded)” in query definition and query v3; or record does not mention outbreak, epidemic, or pandemic; or record does not mention public health, risk to public health, public health emergency, and related concepts [both “acute” and “chronic”]); record does not mention ethics or ethical aspects; and record is not in English

### Screening of Full Texts

We made use of Zotero's automatic download feature to retrieve the full texts of the previously shortlisted records. For papers that were not amenable to automatic downloading, we conducted a manual search to obtain them. For a paper to be incorporated into our main corpus, it had to meet the following inclusion criteria: full text is available; full text mentions social listening or infodemic management (directly or indirectly; see “infodemics (expanded)” in query definition or query v3; or full text mentions outbreak, epidemic, or pandemic; or full text mentions public health, risk to public health, public health emergency, and related concepts [both “acute” and “chronic”]); full text mentions ethics or ethical aspects; and full text is in English

### Paper Assessment

To streamline the process of assessing all retrieved items, we designed a specialized web app leveraging Python (Python Software Foundation) and its Streamlit framework [85]. A comprehensive assessment of the corpus is available for scrutiny via the aforementioned web app or in the study’s OSF repository [83]. This custom-built platform facilitates multiuser access and interaction and is securely hosted on a Firebase (Google LLC) database. We recorded a wide array of details related to each paper, including adherence to the established inclusion criteria; country of origin or focus of the research; year of the study (which might differ from the year of publication); specific health emergency or health-related issue tackled; type of study, whether theoretical, empirical, a literature review, and so on; methodological approach used; in-depth understanding and definition of infodemic management and social listening strategies presented; exploration of ethical considerations concerning infodemics; ethical challenges in infodemic management; aims of infodemic management and social listening strategies; and concluding insights and recommendations.

### Analysis

We conducted an analysis of papers that met our inclusion criteria to evaluate the fundamental themes that emerged from the literature. Our thematic analysis entailed identifying recurring patterns, key concepts, and trends related to ethical considerations in the context of infodemic management and social listening during health emergencies. It is important to note that not all these concepts fit the standard definition of “principle”; some encompass processes and conceptual frameworks that were not previously categorized as principles (see discussion in the *A Methodological Note on our Approach to Ethics* section). In addition to the substantive principles essential for addressing ethical concerns in infodemic management, we have also identified proethical procedural principles [32]. As mentioned earlier, these may not conform strictly to the traditional definition of principles in ethics; they encompass a mix of principles, concepts, and processes. However, when implemented, they ensure adherence to substantive principles and effectively address ethical tensions and issues in infodemic management. To structure our analysis, we created a comprehensive analytical framework that revolved around 2 core areas: the ethical issues pertinent to infodemic management and the ethical aims or values to be pursued in this context. To ensure a systematic approach, we associated these themes with their respective source papers. This phase laid the groundwork for the subsequent synthesis of findings and the development of a profound understanding of the ethical dimensions of infodemic management, as elaborated in the *Results* section. Our coding procedure was conducted in a blinded manner. Initially, 2 independent researchers took detailed notes on ethical issues in infodemic management using a specialized web application built with Python and the Streamlit framework [85]. In the second step, they categorized these issues into thematic clusters based on the output of the application, which presented data in a tabular data set without direct reference to the source papers. Finally, 2 independent researchers collaboratively coded and mapped the different ethical issues and objectives in infodemic management.

## Results

### Characterization of Included Papers

**Figure 1:**
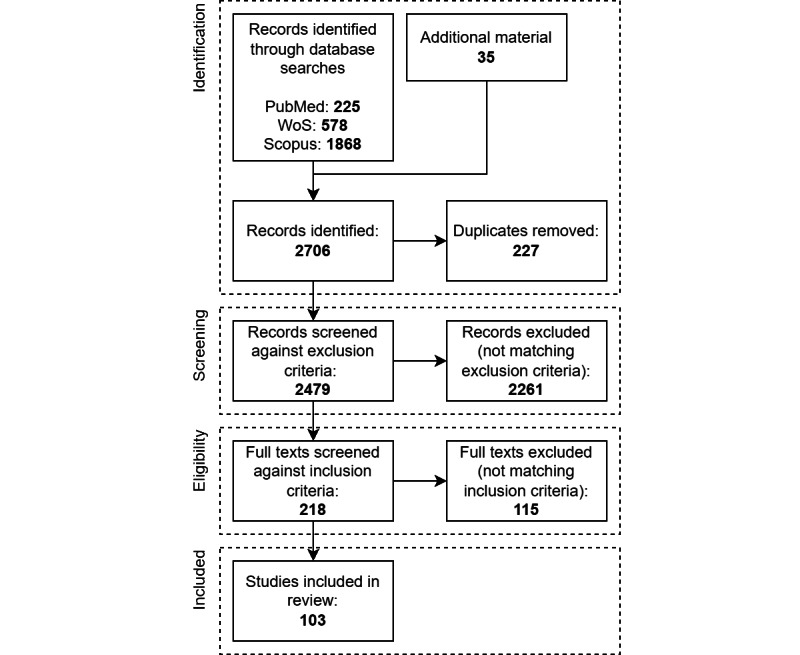
PRISMA (Preferred Reporting Items for Systematic Reviews and Meta-Analyses) diagram.

Through the query described in the *Methods* section of the paper, we identified 225 records through PubMed, 578 through Web of Science, and 1868 through Scopus, as described schematically in the PRISMA diagram in Figure 1. We also manually included 35 additional items, which were considered to be relevant by the WHO EG on the ethics of infodemic management and social listening [29], most of which were published recently and would have otherwise been excluded by our inclusion criteria (ie, papers until the end of 2022). We identified 2706 records and removed 227 (8.4%) duplicates. We screened the remaining 2479 (91.6%) records against the exclusion criteria and excluded 2261 (91.2%) items. Of the remaining 218 full texts, we excluded those that did not match the inclusion criteria; we removed 115 (52.8%) full texts for a total of 103 (47.2%) studies included in the review (Figure 1). Among the studies included in our systematic scoping review, we encountered a diverse array of publication types, including 88 journal papers (85.4%), 9 documents (8.7%), 2 preprints (1.9%), 2 reports (1.9%), 1 presentation (1%), and 1 book (1%). These studies encompassed a spectrum of research types, most of which were theoretical studies (n=45, 43.7%), followed by empirical (n=35, 33.9%), viewpoints or commentaries (n=14, 13.6%), literature reviews (n=4, 3.9%), or other type of studies (n=5, 4.9%). Focusing on empirical research items, most studies were either observational (n=9, 25.7%) or cross-sectional research (n=8, 22.9%), while experimental studies were the least frequent (n=5, 14.3%). The included literature discussed different types of health emergencies. Of the 103 papers, 49 (47.6%) papers explored ethical aspects related to infodemic management and social listening during pandemics and epidemics, including COVID-19, H1N1 influenza, HIV, measles, H5N1, and dengue. An additional 24 (23.3%) of the 103 papers focused on infodemics related to the COVID-19 pandemic or vaccine hesitancy. A few (n=6, 5.8%) papers addressed chronic health emergencies, including those related to smoking, alcohol, obesity, nutrition, and food risk, while others discussed environmental hazards such as radioactivity, floods, disasters, water pollution, and contamination. Of note, most research items (n=70, 68%) were published during the COVID-19 pandemic, with a significant surge from 2020 onward, peaking in 2021 (n=37, 35.9%) and remaining substantial in 2022 (n=18, 17.5%). We also looked at the geographical origin of the papers included in the review. Breaking down the analysis per continent, we found that Europe (n=58, 56.3%) is the most represented continent in our review database, followed by North America (n=38, 36.9%) and Asia (n=27, 26.2%). Only 1 (9.7%) item published by researchers from Central and South America was included (Figure S1 in Multimedia Appendix 1). Since the cutoff was at the end of 2022, we initially made reference to the Open Science Framework repository of 1 paper [82]; however, during review, this paper was published and we decided to link to the published version instead.

### Ethical Issues and Ethical Aims in Infodemic Management and Social Listening

We analyzed the 103 papers included in our review. Noteworthy themes emerged, in particular the relevance of adopting nuanced strategies for effective communication, outreach efforts, and the importance of disseminating truthful information. Trust and the consequences of mistrust in infodemic management contexts were also significant areas of discussion. Furthermore, ethical practices concerning surveillance and social listening were also key points of discussion and, in particular, the ethical implications of social listening practices on privacy rights. In addition, several papers evaluated the importance of addressing vulnerability and equity in the design of infodemic management and social listening strategies, as well as navigating the balance between safeguarding free speech and combating misinformation. Other topics included the lack of community engagement in infodemic management and social listening, the ethical dilemma of informing versus manipulating the public toward desired health behaviors, conflicts of interest, and the importance of honesty, as well as the critical need for education and fostering critical thinking skills to build autonomy. Table 1 provides a comprehensive overview of the identified ethical issues and references.

In addition to exploring ethical issues, we examined the overarching ethical aims of infodemic management and social listening discussed in the corpus of papers. Our analysis revealed primarily the ethical importance of disseminating truthful information. Notably, social listening emerged not only as an issue but also as an ethical goal in infodemic management, that is, listening to and understanding concerns and reacting to them in a timely manner. Furthermore, crucial aims included community engagement, trust-building initiatives, transparency, and educational strategies. In addition, ensuring inclusivity and equity, leveraging fact-checking mechanisms to counter misinformation, and prioritizing privacy and anonymization in social listening practices were among the top aims identified. While many of these ethical aspects were already recognized as crucial ethical issues requiring resolution, their prominence as aims in infodemic management, and not only as issues to solve, highlights the primary importance placed on resolving ethical tensions and integrating ethics into these practices. Table 2 shows a comprehensive list of the identified ethical aims and references.

**Figure 2:**
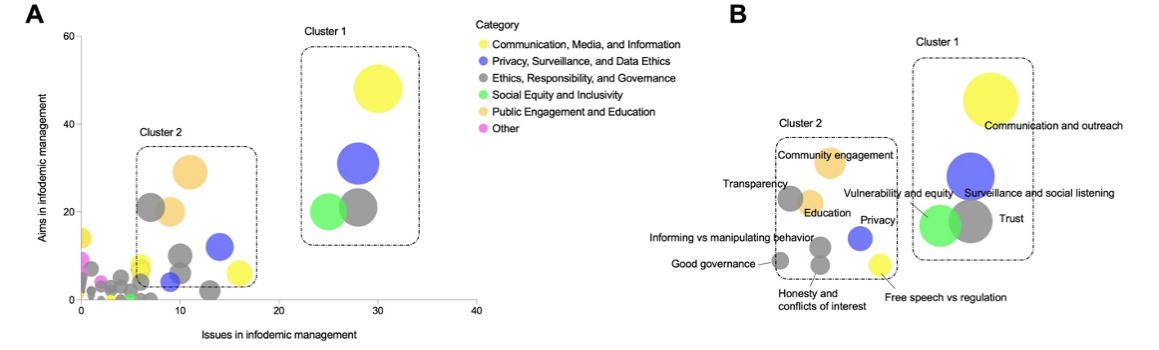
Ethical issues and aims in infodemic management and social listening. The x-axis illustrates the frequency of specific ethical issues discussed in the literature. (A) The y-axis measures the frequency of ethical aims in infodemic management, which is also based on the number of papers discussing them in the analyzed literature. (B) The size of the bubbles represents the sum of the x and y values and serves as a graphical representation of the overall relevance of the ethical aspect or principle under consideration.

To visualize the data, we graphically represented the relevance of the different ethical issues and aims in infodemic management by creating a bubble graph (Figure 2A), in which we defined, on the x-axis, the relevance of each ethical issue based on how often it was discussed in the corpus of publications retrieved with our query. The value on the y-axis is determined by how often each ethical aim was represented in our corpus of publications. The entire list of ethical issues and aims is presented in Multimedia Appendix 2. The size of the bubbles, determined by the sum of the x and y values, visually represents the relevance of the ethical concepts and principles. This serves as a starting point to define which aspects have been considered by the literature when integrating ethical approaches in infodemic management. We also split the different ethical concepts and principles into 6 different categories, as highlighted by the different colors of the bubbles (Figure 2A). The main categories we identified were linked to “communication, media, and information”; “privacy, surveillance, and data ethics”; “ethics, responsibility, and governance”; “social equity and inclusivity”; and “public engagement and education.” We identified 2 relevant clusters housing the most prevalent ethical concepts and principles within our corpus of publications. These clusters will be subject to our analysis in this review, with cluster 1 principles being discussed in the main manuscript and cluster 2 ethical considerations being discussed in Multimedia Appendix 1 (Figure 2B).

**Table 1:** List of ethical issues in infodemic management and social listening and the frequency with which the issue has been reported in the literature analyzed in the review (n=103).

Rank number	Ethical issue in infodemic management and social listening	Papers, n (%)	References
1	Right to be informed truthfully, communication, and outreach	30 (29.1)	[17,18,36-40,45,53,61-64,86-102]
2	Trust and mistrust	28 (27.2)	[16,18,38,40,51,52,57,58,65,66,88,90,91,93,94,96,99-101,103-111]
3	Surveillance and social listening	28 (27.2)	[6,7,16,17,27,36,40,44-50,54,55,60,67-69,86,98,108,112-116]
4	Vulnerability and inequity	25 (24.3)	[36-38,50-53,55,59,63,68,82,87-89,98,101,103,110,113,117-121]
5	Free speech versus regulation	16 (15.5)	[2,7,17,40,42,59,62,64,70,71,82,90,107,110,122,123]
6	Right to privacy	14 (13.6)	[7,47,51,52,54,55,65,69,108,114-117,124]
7	Lack of community engagement	11 (10.7)	[17,18,46,54,57,63,86,98,112,125,126]
8	Informing versus manipulating	10 (9.7)	[17,36,40,61,90,97,104,110,127,128]
9	Honesty and conflicts of interest	10 (9.7)	[36,38,39,51,63,71,105,109,126,129]
10	Lack of education	9 (8.7)	[17,36,46,62,88,96,110,123,130]
11	Necessity	9 (8.7)	[17,49,61,63,68,86,92,105,126]
12	Cybersecurity	9 (8.7)	[7,36,52,54,65,82,98,108,124]
13	Lack of transparency	7 (6.8)	[36,38,54,63,86,92,93]
14	Individual versus collective health	7 (6.8)	[51,58,59,61,94,109,122]
15	Good governance	6 (5.8)	[7,39,60,63,71,129]
16	Epistemic underdetermination	6 (5.8)	[39,61,63,92,96,99]
17	Lack of autonomy	6 (5.8)	[52,59,61,62,104,121]
18	Power imbalances	6 (5.8)	[38,58,59,97,100,103]
19	Translation of evidence into public health practice	6 (5.8)	[63,71,96,98,99,110]
20	Responsibility	5 (4.8)	[58,62,105,109,131]
21	Different cultural perspectives	5 (4.8)	[41,51,87,96,126]
22	Stigma	4 (3.9)	[109,119,120,123]
23	Definition of truth	4 (3.9)	[2,62,71,99]
24	Alignment with human rights framework	4 (3.9)	[86,89,121,122]
25	Legality	4 (3.9)	[69,112,123,131]
26	Proportionality	4 (3.9)	[61,93,112,126]
27	Social media practices	3 (2.9)	[42,129,130]
28	Control of citizens	3 (2.9)	[54,60,64]
29	Selection bias and information bias	3 (2.9)	[2,17,66]
30	Fairness	3 (2.9)	[53,109,126]
31	Appeal to fear	3 (2.9)	[102,109,127]
32	Data and representation inclusiveness	3 (2.9)	[44,98,113]
33	Lack of research	2 (1.9)	[28,98]
34	Beneficence	2 (1.9)	[42,89]
35	Solidarity	2 (1.9)	[52,63]
36	Lack of openness	1 (1)	[48]
37	Criminalization	1 (1)	[70]
38	Lack of independent oversight	1 (1)	[112]
39	Reciprocity	1 (1)	[63]
40	Absence of an ethical framework	1 (1)	[86]

**Table 2:** List of ethical aims of infodemic management and social listening and the frequency with which the aims have been reported in the literature analyzed in the review (n=103).

Rank number	Ethical aims in infodemic management and social listening	Papers, n (%)	References
1	Truthful communication and outreach	48 (46.6)	[6, 16-18, 27, 28, 36, 39, 43-45, 47, 48, 50, 54, 55, 57, 60-64, 69, 71, 82, 86, 87, 88, 92, 95, 100, 104, 107, 117-119, 123-125, 127, 132-139]
2	Surveillance and social listening	31 (30.1)	[6, 7, 16, 17, 27, 36, 37, 43, 44, 47, 50, 51, 53, 54, 59, 60, 82, 86, 87, 96, 98, 108, 113, 117, 123, 124, 128, 130, 131, 134, 136]
3	Community engagement	29 (28.2)	[16,17,41,43-47,50,53,55,60-63,67,82,86,96,112,114,117,125,133-136,140,141]
4	Trust	21 (20.4)	[7,17,28,36,43-45,57,61,66,70,82,106,108,117,132,133,135,139,141,142]
5	Transparency	21 (20.4)	[7,17,41,43-45,48,50,61-63,72,112-114,119,124,134,139-141]
6	Empowerment through education and educational strategies	20 (19.4)	[16,27,28,42,47,55,57,59,62-64,70-72,82,96,106,118,128,138]
7	Inclusivity and equity	20 (19.4)	[6,17,18,28,37,44,50,53,55,61-63,67,72,82,92,98,117,138,139]
8	Effectiveness of targeted interventions	14 (13.6)	[17,36,39,45,50,60,61,63,112,119,127,132,135,141]
9	Fact checking and labeling misinformation and debunking misinformation	14 (13.6)	[6,27,36,37,62,70,104,105,110,118,121,123,124,128]
10	Guarantee privacy and anonymization	12 (11.6)	[7,17,48-50,60,67,68,112,114,116,124]
11	Influencing health behavior and improving health	10 (9.7)	[52,59,61,64,82,92,96,97,100,117]
12	Foster cooperation between institutions	9 (8.7)	[41,43,45,50,55,72,117,124,136]
13	Transform evidence in communication and policies	8 (7.8)	[16,17,47,55,61,112,134,137]
14	Openness	7 (6.8)	[17,50,54,60,86,134,141]
15	Good governance	7 (6.8)	[6,7,39,44,50,63,64]
16	Honesty and integrity	6 (5.8)	[27,39,45,61,63,134]
17	Acknowledgment of failure and evaluation of impact	6 (5.8)	[43-45,51,69,143]
18	Acknowledging uncertainty (epistemic underdetermination)	6 (5.8)	[37,43,44,63,134,143]
19	Respect for human rights	5 (4.8)	[45,82,112,114,134]
20	Respect for dignity and persons	5 (4.8)	[45,49,50,109,112]
21	Research and generation of new ideas	4 (3.9)	[6,45,57,125]
22	Cybersecurity	4 (3.9)	[7,49,50,124]
23	Justice	4 (3.9)	[17,59,107,143]
24	Autonomy	4 (3.9)	[17,45,49,50]
25	Blocking and removing misinformation and conspiracy theories	4 (3.9)	[36,37,40,93]
26	Accountability	3 (2.9)	[45,72,112]
27	Nondiscrimination and stigma	3 (2.9)	[61,109,112]
28	Solidarity	3 (2.9)	[63,139,143]
29	Population tracing and control	3 (2.9)	[54,98,136]
30	Proportionality	2 (1.9)	[63,139]
31	Using ethical approaches (ethics for ethics)	2 (1.9)	[36,140]
32	Guaranteeing free speech	2 (1.9)	[71,118]
33	Fairness	2 (1.9)	[17,45]
34	Independent oversight	2 (1.9)	[67,72]
35	Improve society, social cohesion, reduce polarization	2 (1.9)	[66,82]
36	Responsibility	2 (1.9)	[45,95]
37	Stewardship	2 (1.9)	[61,139]
38	Enforcement of recommendations and restrictions	2 (1.9)	[52,94]
39	No harm	1 (1)	[112]
40	Acknowledge the limitations of social listening practices	1 (1)	[44]
41	Reciprocity	1 (1)	[63]
42	Protect health care professionals	1 (1)	[42]
43	Maintenance of peace	1 (1)	[107]
44	Maintenance of democracy	1 (1)	[107]
45	Collect data into a single and accessible platform	1 (1)	[69]

### Analysis and Application for Each Principle

In this second part of the *Results* section, we will introduce each concept and principle identified in cluster 1 (Figure 2B) and define, for each, which specific ethical issues emerged from the literature, which specific associated aims and goals should be achieved, and why these aims should be achieved in the context of infodemic management. For each of these concepts and principles, we will identify procedural principles, which, if applied to infodemic management, can provide practical guidance and recommendations on how to ensure that substantive underlying ethical principles are respected while safeguarding the effectiveness of infodemic management practices.

### Truthful Communication and Outreach

The systematic scoping review encompassed a comprehensive examination of literature pertaining to communication and outreach in the context of infodemic management and the practice of social listening. Several critical findings emerged, and they were categorized into distinct thematic aspects, each of which holds significant implications for ethical and effective communication and outreach in the context of infodemic management.

The first pertains to inclusivity. The reviewed literature emphasizes the importance of crafting communication strategies that take into account the needs of vulnerable groups [36,86,87]. This includes individuals with limited or no access to the internet or with restricted use of social media platforms, such as those who rely solely on services such as WhatsApp for information. Recognizing the fragility of communication technologies is paramount, as technical disruptions can impede information dissemination efforts [37]. In particular, the literature highlights the vulnerability of individuals with low information and media literacy, who are at a heightened risk of falling victim to misinformation [88]. Consequently, the ethical principle of vulnerability intersects with the imperative for education and literacy. It is therefore fundamental to address these vulnerabilities in communication strategies [38,89].

The second thematic aspect underscores the significance of maintaining consistency and reliability in information dissemination to foster public trust [88]. Addressing information gaps and uncertainties is crucial to mitigate the spread of misinformation [90]. However, it is essential to exercise caution when providing information in situations characterized by epistemic underdetermination [39,91,92]. Incorrect or imprecise information can have detrimental effects, contributing to information overload and confusion among recipients [40]. This can erode institutional trust and hinder the receptivity of future public health advice [18]. Therefore, a foundation of evidence-based and epistemically truthful communication is advocated to guide the development of public health messages and strategies, which ultimately serve to bolster public trust [93].

Furthermore, the literature emphasizes the risk of information overload, even when the information is accurate. To mitigate this, communication from reliable sources should strike a balance between countering disinformation and avoiding overwhelming the intended audience. Information should be timely, accurate, disseminated through appropriate channels, and designed for the specific target population [94]. Ensuring clarity and timeliness is considered fundamental, and the use of plain language and suitable metaphors is recommended to enhance public comprehension [16,144]. The employment of personnel experienced in scientific communication can be instrumental in conveying complex scientific information to the public. Tailoring messages to specific audiences significantly improves understanding and engagement, especially in risk and crisis communication situations, where reassurance and panic mitigation are integral strategies [17].

A third aspect identified in the literature centers on the dynamics of social media communication as a key component in infodemic management [145,146]. Social media platforms are recognized as significant channels for information dissemination, making social media literacy an essential skill for effective communication [147]. The choice of communication channels should align with the specific message being conveyed and the intended target public [17]. Engaging with influencers, key opinion leaders, and religious figures is seen as a means to aid in the dissemination of accurate information [17,41,145].

Importantly, the reviewed literature underscores the importance of improving the information ecosystem as a fundamental goal in infodemic management. This involves enhancing access and exposure to credible health information and encouraging positive changes in information-seeking behavior [18,92,95,96]. Promoting information literacy among the public empowers individuals to critically evaluate and understand the information they encounter, thereby reducing the impact of misinformation [17,42].

Finally, the literature stresses the necessity of continuously assessing the effectiveness of communication strategies. Feedback should inform the development of new and improved approaches. In this context, advocacy and community engagement are recognized as pivotal in ensuring effective communication and outreach [43,44,97]. Involving representatives of the target public in the design of communication strategies enhances effectiveness and ensures that community voices are heard [45,46]. Small-scale social listening approaches, in which community members provide real-time feedback, offer valuable insights into the effectiveness of communication and outreach strategies [43,44]. These elements collectively contribute to the efficacy of public health measures and ensure that communication is conducted in an ethical and responsible manner.

**Figure 3:**
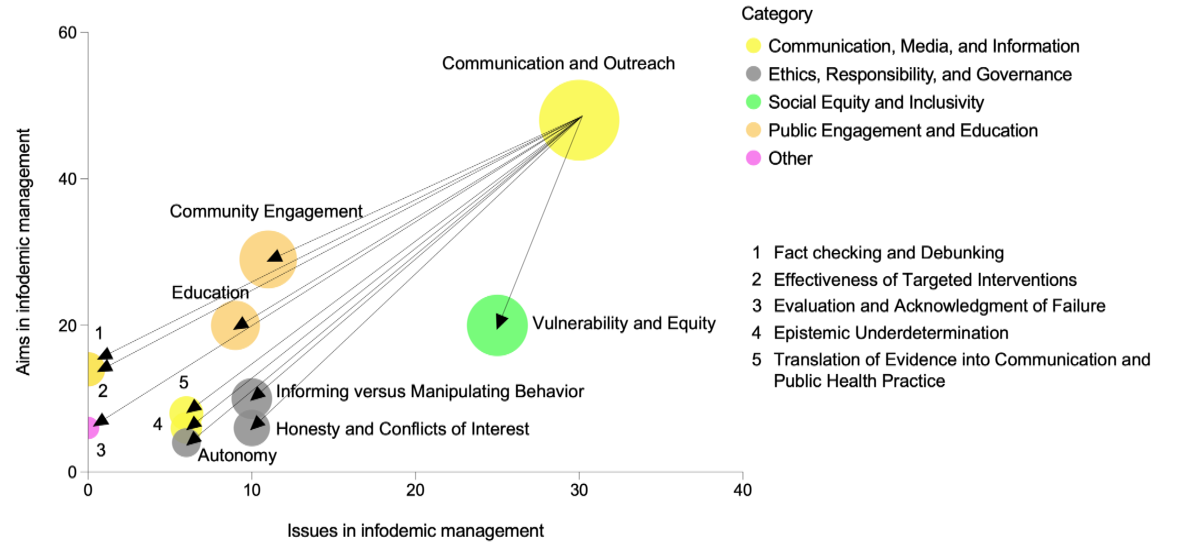
Procedural principles to ensure ethical and effective communication and outreach for infodemic management. The x-axis illustrates the frequency of specific ethical issues being discussed in the literature. The y-axis measures the frequency of ethical aims in infodemic management, which is also based on the number of papers discussing them in the analyzed literature. The size of the bubbles represents the sum of the x and y values and serves as a graphical representation of the overall relevance of the ethical aspect or principle under consideration.

In light of the insights and considerations drawn from the reviewed literature, we have articulated a set of ethical procedural principles. When applied to communication and outreach within the framework of infodemic management practices, these principles enhance their effectiveness while upholding ethical standards, as visually represented in Figure 3.

The first principle, encapsulated by the terms *vulnerability and equity*, underscores the imperative of ensuring equitable access to information, particularly for vulnerable groups, thus promoting inclusivity. Another critical facet is *community engagement*, emphasizing the active involvement of the public in infodemic management and social listening practices. This involvement, notably in the design of communication strategies and the generation of feedback, facilitates the integration of public concerns into public health initiatives, whether related to prevention or risk and crisis communication [17]. Empowerment through *education* serves as a substantive ethical requirement, emphasizing the significance of fostering information literacy to nurture a healthier information ecosystem and mitigate the adverse effects of misinformation. The concept of *epistemic underdetermination* acknowledges the need to address information gaps transparently, especially when evidence-based information is unavailable. In Figure 3, the issue of *informing versus manipulating behavior* intends to highlight that a highly literate health information ecosystem should provide adequate information to enhance individual and public health without resorting to manipulative tactics, which could erode institutional trust over time [18,88,127]. The principles of honesty and avoiding conflicts of interest, represented as *honesty and conflicts of interest* in Figure 3, emphasize the pivotal role of honesty and the avoidance of conflicts of interest in shaping communication and outreach within infodemic management, thereby safeguarding trust. The principle of *autonomy* relates to a combination of building information literacy through educational approaches and ensuring inclusivity and equity. The principle of *evaluating and acknowledging failure*, presented in Figure 3, encourages the development of more effective information campaigns through iterative processes involving communities. *Effectiveness of targeted interventions* underscores that ethically sound communication campaigns are a prerequisite for their effectiveness. *Fact checking and debunking*, presented in Figure 3, further underscores the importance of these activities as primary objectives in communication and outreach efforts to combat misinformation. Finally, *translating evidence into public health practices* emphasizes the critical task of translating evidence into tangible health benefits for individuals and communities, even in the face of challenges such as epistemic underdetermination or a polarized and misinformation-rich information ecosystem.

These ethical procedural principles together constitute a robust foundation for the development and implementation of communication and outreach strategies within the context of infodemic management. Their application not only bolsters the effectiveness of these practices but also ensures ethical integrity and adherence to ethical standards.

### Monitoring and Social Listening

We also examined the landscape of ethical considerations regarding monitoring and social listening in infodemic management. This comprehensive exploration has revealed several pivotal findings and strategic approaches, each bearing profound implications for the ethical conduct of monitoring and social listening as they relate to the management of information epidemics.

In the ethically complex arena of surveillance and data collection, under specific circumstances requiring comprehensive data sets and rigorous data protection, individuals may have an ethical obligation to contribute to monitoring even without explicit consent [47-50]. That said, the principle of autonomy, implemented through the practice of obtaining informed consent, should stand as the foremost pillar whenever possible. It advocates for integrating informed consent into social listening practices, ensuring that individuals are aware of and consent to data collection [47]. The literature widely advocates for informed consent as a key ethical practice in data collection, emphasizing that it is essential, not a hindrance, in sustaining institutional trust and respecting privacy rights [47,48,50]. This approach enhances public confidence in data-driven methods. Balancing data and privacy, raising privacy awareness, and maintaining confidentiality and anonymity are crucial for upholding the ethical standards of data collection and therefore mitigating the erosion of trust [51,52].

Transparency is the bedrock upon which trust is constructed. To foster public trust and ethical conduct, articulating data collection practices to the public and offering a clear overview of the social listening strategy is suggested to be fundamental in the analyzed corpus of literature [38,86,93]. This transparency assures the public that their data are managed responsibly. A cardinal rule in ethical data collection is to prioritize *active* social listening practices over *passive* social listening approaches [38,48]. Engaging with the public actively, considering their concerns, and respecting their autonomy are in line with key ethical principles. Instead, passively extracting data, for example, to monitor public concerns and rumors, may impact public trust in the medium and long term [17,38,48]. The corpus of literature thus underlines that ethical decision-making demands that all public concerns are taken into account when converting social listening insights into infodemic management strategies [14,112]. Community engagement is not just an ethical necessity but a source of valuable insights [53,148,149].

Furthermore, the literature underlines that it is crucial to emphasize that social listening must not be used for tracking dissent, population control, or governmental monitoring [48,54]. This ethical use safeguards against potential misuse and violations of privacy [50,54,55].

A second important aspect to ensure ethical data collection is that representativeness in data is the standard. This inclusivity encompasses demographic diversity, language considerations, and the types of data captured [44,87,98,113]. By avoiding research biases, ethical data collection becomes a more powerful tool for understanding the information landscape [17,44]. If the design of social listening is not inclusive, the conclusions drawn from the data may have limited or even negative repercussions for vulnerable groups or groups that were not considered or integrated into the social listening design [14,44].

To ensure effective social listening, monitoring information should happen in real time, including both online and offline sources. This is instrumental in detecting narratives, questions, concerns, and misinformation within the information ecosystem. Despite the focus on effectiveness, data security remains paramount, ensuring the protection of sensitive data and preventing unauthorized access or use [53,54,148,149]. Evidence suggests that a breach in security would lead to a reduction of trust, and a reduction of trust would lead to decreased effectiveness of social listening [54]. Thus, also in this case, ethical social listening is necessary to guarantee the effectiveness of infodemic management practices in the short and long term. In addition to cybersecurity, respecting individuals’ right to privacy is considered fundamental [50,54,55]. This includes data anonymization and the implementation of robust data security measures to safeguard sensitive information. When anonymization and data security are guaranteed, in situations of necessity, social listening practices can shift more toward passive approaches [17,48,54].

**Figure 4:**
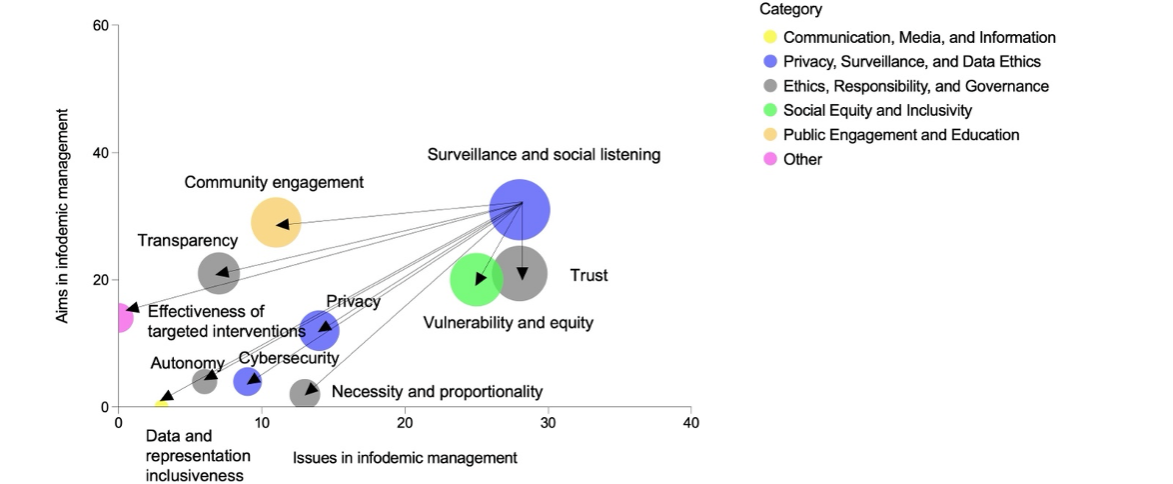
Procedural principles to ensure ethical and effective social listening practices. The x-axis illustrates the frequency of specific ethical issues discussed in the literature. The y-axis measures the frequency of ethical aims in infodemic management, which is also based on the number of papers discussing them in the analyzed literature. The size of the bubbles represents the sum of the x and y values and serves as a graphical representation of the overall relevance of the ethical aspect or principle under consideration.

In line with the methodology outlined in our communication and outreach framework and guided by the recommendations and insights gleaned from the reviewed literature, we have formulated a set of ethical procedural principles applied to the domains of surveillance and social listening; these principles are instrumental in bolstering the efficacy of social listening practices while upholding the highest ethical standards, as visually depicted in Figure 4.

The most important procedural principle is *trust*. Prioritizing trust fosters the perception of surveillance and social listening endeavors as constructive measures dedicated to ensuring public health. The second principle emphasizes inclusivity and equity (Figure 4; *vulnerability and equity*), advocating for the development of social listening practices that are free from biases and sensitive to the needs of vulnerable populations. It is imperative that the design and implementation of these practices aim for the utmost representation, extending to the insights derived from social listening (*data and representation inclusiveness* as another procedural principle). *Community engagement* is the third aspect to consider, suggesting the preference for the adoption of *active* social listening strategies that directly involve the target audience, thereby cultivating trust in the processes. *Privacy* and *cybersecurity* constitute 2 fundamental principles; these principles demand that privacy and anonymity be guaranteed, especially when *passive* social listening methods are used. In addition, *transparency* is another procedural principle in this context, closely linked to trust and requiring clear communication of the purpose behind any social listening action to dispel any negative perceptions held by the public. *Necessity and proportionality* are key operational principles in this context. To avoid invasiveness in social listening, it is essential to ensure that such practices are only used when absolutely necessary and in proportion to the specific circumstances [50]. *Autonomy* is also considered a principle to guarantee ethically sound and effective surveillance and social listening. It underscores the importance of ensuring that the target audience comprehends the practices they are subject to and that they have the power to assert control over their own privacy, cybersecurity, and personal information.

### Trust and Mistrust

At the core of trust building lies the imperative to involve the public actively [17,38], steering clear of top-down approaches; this is especially valid for health departments and governmental entities [17,38,150]. Collaborative decision-making and involving the public in shaping policies engender a sense of inclusivity and shared responsibility [18]. Furthermore, discouraging the pursuit of profit-driven objectives in public health is crucial. Rather, decisions and actions should be grounded in a commitment to the well-being of the public, prioritizing their welfare above all else [37,56].

The literature further highlights that a critical building block for trust building is the elevation of trust in research and researchers [57,93,103]. This involves ensuring that research is conducted ethically, findings are communicated transparently, and public health initiatives are rooted in sound scientific evidence. Ethical communication principles are paramount, emphasizing the importance of not resorting to manipulative tactics, even when pursuing noble causes [90,104]. Informed and transparent communication with the target public is essential, promoting honesty and avoiding any perception of manipulation (for a detailed explanation, refer to the *Truthful Communication and Outreach* section) [38,93].

Furthermore, another important aspect of trust is its connection to credibility. Trust is bolstered by credibility and the use of expertise. Establishing oneself (this, for example, includes researchers, public health institutions, and health departments within governments) as a reliable and knowledgeable source of information is pivotal in instilling trust in public health communications [58,114,117].

Another important aspect in building trust is the engagement of various public groups as coactors in the planning and execution of infodemic management and social listening initiatives; this is not merely an ethical requirement but also provides a wealth of valuable insights that ensure the effectiveness of infodemic management and social listening actions. A pluralistic approach ensures that all voices are heard and considered in the decision-making process [17,18,51,57].

Finally, the literature suggests that even public health institutions, which have no financial interests, can benefit from adopting branding and advertising strategies similar to those used by business-oriented organizations. This approach helps to effectively highlight the services they offer to the public. This strategic approach can contribute to a stronger and more recognizable public health identity. These strategic solutions collectively form a robust framework for enhancing trust and countering mistrust within the domain of public health, and that indirectly reflect on trust for infodemic management and social listening practices performed by such institutions [16,17].

**Figure 5:**
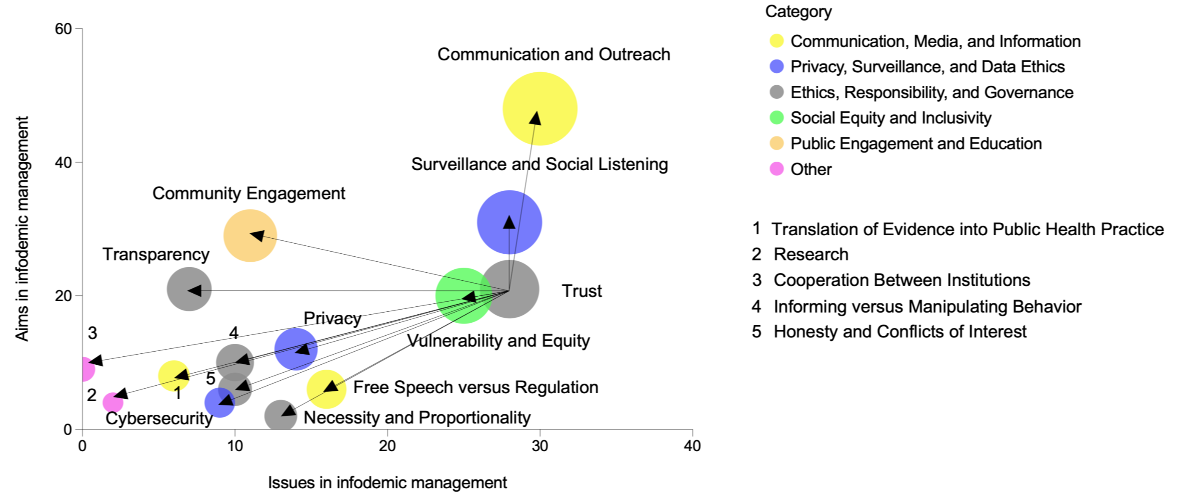
Procedural principles to build trust and reduce mistrust. The x-axis illustrates the frequency of specific ethical issues discussed in the literature. The y-axis measures the frequency of ethical aims in infodemic management, which is also based on the number of papers discussing them in the analyzed literature. The size of the bubbles represents the sum of the x and y values and serves as a graphical representation of the overall relevance of the ethical aspect or principle under consideration.

Trust appears to be a key core principle to ensure the effectiveness of infodemic management and social listening in the medium- and long-term horizons, closely linked to several procedural principles as shown in Figure 5. By integrating these principles into practice, thus strengthening trust, public health efforts through infodemic management can be not only more deeply rooted in ethical conduct but also more effective.

As highlighted by the procedural principles for trust in Figure 5, at its core, trust is nurtured through transparent, informed communication, free from manipulation [38,90,93,104]. This substantive principle ensures that the public receives accurate information. *Privacy* and *cybersecurity* principles, as highlighted in Figure 5, are nonnegotiable, safeguarding data protection and personal privacy within surveillance and social listening. Upholding these ethical standards reduces the likelihood of developing public mistrust in the institution carrying on infodemic management and social listening practices. Furthermore, *community engagement* ensures that the voices of the community are actively incorporated into the decision-making process. In fact, building trust involves embracing inclusive approaches, and similarly, it involves addressing the needs of vulnerable populations. Left out and marginalized voices that are not considered by the institutions leading infodemic management and social listening efforts would lead to increased mistrust. Similarly, adhering to the principles of *necessity and proportionality* is key to justifying the invasive nature of these practices and maintaining trust. The literature also identified *transparency* as a key procedural principle in this context: by explaining the purpose of social listening practices and dispelling doubts and misconceptions, transparency helps mitigate mistrust [38,93]. Another important aspect considered in the literature is that striking the right balance between free speech and necessary regulation is crucial, preventing overreaching censorship that could erode trust [17,18,59] (*free speech vs regulation* in Figure 5). Furthermore, aligning policies and communications with the existing evidence is essential, as is building trust in research and fostering collaboration between researchers and public institutions [57,93,103] (*translation of evidence into communication and public health practice* and *cooperation between institutions* in Figure 5). It is vital to clarify that communication and policies are designed to inform rather than manipulate, emphasizing the ethical intent behind these practices [90,104] (*informing vs manipulating behavior* in Figure 5). Finally, honesty and the disclosure of any conflicts of interest at every step of infodemic management help guarantee transparency and ensure that the decision-making process remains free from bias or manipulation, enhancing trust and overall effectiveness [38,93,105] (*honesty and conflicts of interest* in Figure 5).

### Vulnerability, Equity, and Inclusivity

The first thematic aspect emphasizes the need to strengthen vulnerable media information ecosystems. To do so, the body of literature suggests that it is crucial to empower individual members of the public and communities to be autonomous and resilient against manipulation tactics [16,122]. This entails strategies to enhance critical thinking; media literacy; and the ability to discern reliable sources, especially among those who are most susceptible to manipulation [17,88,103].

The second thematic aspect underscores the importance of combating polarization within the information ecosystem. The detrimental effects of epistemic echo chambers and bubbles must be mitigated to ensure that all individuals, regardless of their background or beliefs, have access to a balanced and diverse information landscape [16]. Beyond information, the literature advocates for holistic improvements in socioeconomic, cultural, environmental, and “infospherical” conditions [39]. This includes addressing disparities in living and working conditions, access to water and sanitation, housing, education, health care services, and food production. It extends to fostering inclusive social, community, and web-based networks, considering individual lifestyle factors, age, sex, and genetics [17,39]. Equity in these areas is essential to reduce vulnerabilities and is a primary goal of infodemic management. Similarly, infodemic management efforts should focus on improving access to information. Ensuring an equal distribution of such resources among all segments of the population, regardless of socioeconomic status, is paramount in promoting equity and reducing vulnerabilities [53,55,60,151].

The third aspect highlights the monitoring of ethnically targeted disinformation and misinformation that exploits the fears of vulnerable groups, including older adults, leading to mental health burdens [36,59]. Accurate and inclusive social listening designs, alongside proactive measures, are needed to prevent and counteract such disinformation, protecting the most vulnerable groups. Similarly, the literature highlights the importance of preventing information-related discrimination based on ethnicity, religion, or political beliefs. Equitable access to information must be safeguarded for all without discrimination.

The fourth and final thematic aspect that we identified underscores the fundamental right to receive accurate health information. Equitable access to accurate health information is essential for everyone, regardless of their background or circumstances. This requires the establishment of adequate and fair communication channels that cater to different segments of the public, with special attention to minority and vulnerable groups. These channels should ensure that information is accessible and comprehensible [36,38,86,87,89].

**Figure 6:**
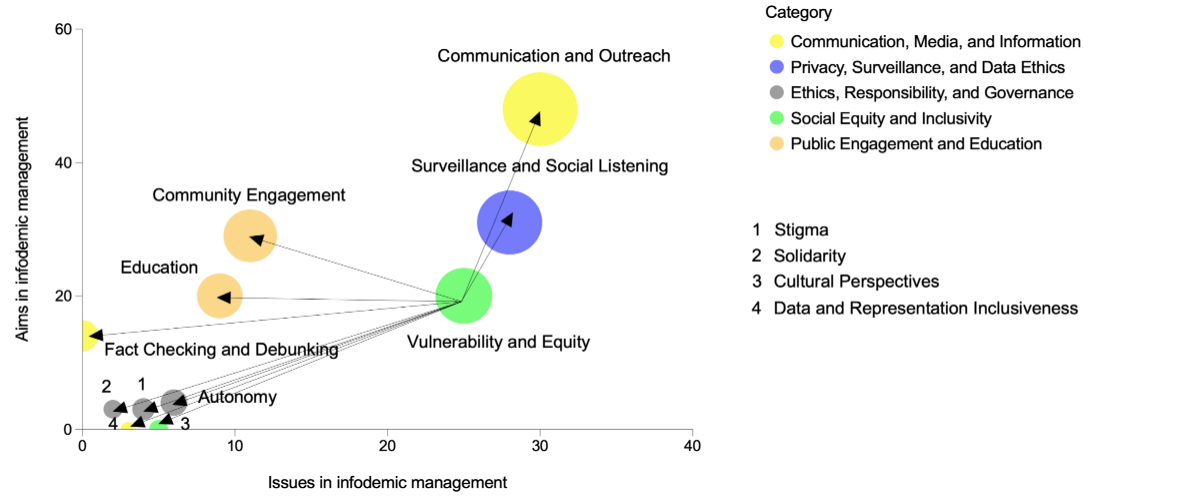
Procedural principles to ensure inclusivity and equity and address vulnerability. The x-axis illustrates the frequency of specific ethical issues discussed in the literature. The y-axis measures the frequency of ethical aims in infodemic management, which is also based on the number of papers discussing them in the analyzed literature. The size of the bubbles represents the sum of the x and y values and serves as a graphical representation of the overall relevance of the ethical aspect or principle under consideration.

On the basis of the recommendations and insights from the reviewed literature, we have formulated a set of ethical procedural principles applied to infodemic management; these principles are instrumental in upholding the highest ethical standards during infodemic management actions, as represented in Figure 6.

The principles of inclusivity and equity are interwoven with various procedural principles that help ensure their application. A holistic approach to inclusivity and equity is pivotal. Inclusive *communication and outreach* strategies must ensure that all voices, with particular emphasis on vulnerable groups, are not only heard but also actively integrated into the decision-making process. Inclusive social listening design is paramount [16,44] (*surveillance and social listening* in Figure 6), demanding that the concerns and perspectives of vulnerable populations be central to the process. *Community engagement* becomes an important bridge, actively incorporating diverse voices in social listening design, decision-making, and communication efforts. Equally important is the drive to reduce vulnerabilities through *education* and literacy, especially among the marginalized groups [17,88,103]. The battle against misinformation and disinformation targeting vulnerable groups within polarized information ecosystems thus becomes an ethical imperative. In this context, *autonomy* should be granted, empowering individuals to protect themselves from the dangers of disinformation. Furthermore, addressing *stigma* is critical, ensuring that no one is excluded from social listening initiatives. In this context, *solidarity* serves as the moral backbone, affirming that all individuals are equally valued [16]. Importantly, adapting to various *cultural perspectives* is important; that is, infodemic management actions need to be tailored to different cultural perspectives, recognizing that infodemic management is not a one-size-fits-all practice [60]. Finally, *data and representation inclusiveness* guarantees that the concerns of vulnerable groups are acknowledged and responded to, fostering equity and inclusivity [17]. This entails ensuring that insights from infodemic management reports incorporate the concerns of vulnerable groups and propose tailored solutions.

### Principles in Cluster 2

In this cluster, we identified and discussed the most represented substantive principles in cluster 1 (Figure 2B) in the analyzed body of literature, discussed the solutions proposed by the literature to implement and follow them in infodemic management practices, and defined procedural principles that help to ensure that such substantive principles are integrated into infodemic management practices. In Multimedia Appendix 1, we delve into the detailed analysis of principles highlighted in cluster 2 (Figure 2B). These ethical principles are *community engagement*, empowerment through *education*, *transparency*, *free speech versus regulation*, *informing versus manipulating behavior*, *honesty and conflicts of interest*, and *good governance*.

## Discussion

### The Implications of the Findings

The review has illuminated various crucial ethical considerations that can be instrumental in enhancing the effectiveness of infodemic management. The literature surveyed predominantly emanates from the backdrop of the COVID-19 pandemic. This temporal context is both a strength and a weakness: on the positive side, it signifies a wealth of learnings from a recent global crisis that has driven substantial advances in infodemic management strategies; however, it implies that the ethical readiness for infodemics lacked a solid foundation in evidence. Scientific support for ethics within infodemic management was not yet accessible based on the lessons learned before the COVID-19 pandemic and existing literature. It is promising to see a few studies extending their gaze beyond acute health events to investigate chronic health issues. Still, the field must continuously adapt and evolve as new challenges emerge. In general, the literature on infodemic management still lacks a robust foundation in ethics and ethical considerations. This observation highlights the importance of advocating for expanded research efforts in this domain.

The literature indicates a limited number of experimental empirical approaches within ethics in infodemic management. Only a few studies have taken this approach [51,58,87,91,97], underlining the need for more work of this kind. This limitation is particularly concerning, as empirical research is vital for the improvement of infodemic management strategies, serving as the foundation for prevention and preparedness in the face of future infodemics [152]. Another evident limitation of the existing literature is the dominance of Western approaches to ethics in infodemic management. To ensure a comprehensive understanding and inclusivity in ethical considerations, this Western-centric bias should be addressed by incorporating diverse global ethical perspectives [16].

As highlighted by the literature reviewed in this study, it is paramount to emphasize that ethics is not a hindrance but a tool for enhancing the effectiveness of infodemic management and social listening. The review underscored that ethical considerations are instrumental for achieving medium- and long-term effectiveness in these practices.

A few key ethical aspects have emerged as fundamental for different practices linked to infodemic management and social listening. The first is community engagement, which emerged as a central procedural principle, enhancing the quality and effectiveness of communication, surveillance, and social listening efforts. It not only fosters trust in the institutions carrying out these activities but also contributes to improving the strategies themselves through feedback mechanisms [17].

Second, empowering individuals through educational approaches was identified as a fundamental procedural principle. Education, that is, information and media literacy, equips them with the ability to discern between accurate and inaccurate information [18,88,127]. When facing educated and literate publics, institutions that conduct infodemic management activities need to rely less on censorship or manipulative communication strategies, which are detrimental in the medium and long run [110]. Ethical strategies involving empowerment through education, resilience building, and autonomy are thus vital for the efficacy of infodemic management at all stages [62,110].

Third, the importance of inclusivity and equity extends beyond beneficence; it is integral to effectiveness. This is directly connected to community engagement, ensuring that vulnerable individuals and communities have a voice in the design of infodemic management and social listening strategies improves their effectiveness and helps prevent the formation of pockets of polarized resistance to public health communication [17,45,46]. This inclusivity also applies to minority groups holding opinions that do not reflect science-based evidence, such as antivaxxers; engaging with these groups and listening to their concerns are essential for ethical and effective infodemic management and social listening strategies [48]. Of note, engaging and listening to the concerns of these groups does not imply embracing, endorsing, or justifying their opinions [17,43,44,51,57].

Finally, central to all the principles discussed above is trust. Trust is fundamental in ensuring the publics’ receptivity to public health communication and the willingness to share data for social listening purposes. The literature emphasizes that trust plays a crucial role in minimizing the negative effects of information received by the public when such information, albeit being accurate and designed to promote individual and public health, is regarded as manipulative, conspiratorial, and biased toward the interest of the institution that is performing infodemic management activities [90,104,110]. Trustworthy institutions disseminating public health messages encounter less resistance and can leverage the publics’ sense of responsibility [18,62,105]. This concept has implications that could extend beyond infodemic management and social listening, possibly impacting democracy and peace, since trustworthy institutions are thriving in nonpolarized information ecosystems [9]. While these aspects are currently underexplored in the literature, we advocate for further exploration of the potential far-reaching effects of maintaining a healthy information ecosystem with trusted actors and educated, autonomous publics.

Some themes and ethical principles remain underrepresented in the literature included in this review. These aspects should not be necessarily considered as less relevant per se in the context of infodemic management since the literature included in this study only represents the views of the research community limited to the period and data taken into consideration by this systematic scoping review; this underrepresentation should rather highlight opportunities for further research and reflection in the field. For example, independent oversight of ethical infodemic management and social listening practices ensures that none of these practices are conducted in unnecessary situations, without considerations about their proportionality [112], valuing transparency and preventing conflicts of interest [36,38]; all these aspects ensure the maintenance or enhancement of institutional trust. A second example is the integration of different cultural perspectives in infodemic management, circling back to the importance of inclusivity and equity since some of the highlighted procedural principles that enhance the effectiveness of infodemic management may not hold the same value and importance in different cultural contexts [46,51]. These underexplored areas should not be underestimated in terms of their ethical significance and potential impact on infodemic management and social listening effectiveness.

In sum, this systematic scoping review provides a comprehensive understanding of the ethical dimensions of infodemic management. It highlights the critical role of ethics in enhancing the effectiveness of these practices and underscores the need for an ethically and empirically informed approach to infodemics. The findings and principles identified in this review are integral to the continuous improvement and adaptation of strategies for tackling infodemics and safeguarding public health. These findings serve as a foundational element for structuring a WHO ethics guidance and a practical implementation framework on the ethics of infodemic management and social listening, which aims to combine learned lessons from the literature and know-how and expert opinions from a WHO EG on ethical considerations in infodemic management and social listening [29].

### Limitations

While our systematic scoping review offers valuable insights into the ethical dimensions of infodemic management, it is essential to acknowledge certain limitations that shape the scope and generalizability of our findings. First, our review is based on the literature published between 2002 and 2022 (although it includes a few papers and documents contributed by the WHO EG on infodemic management and social listening published after 2022), thereby excluding potentially relevant studies published before or after our cutoff date. Of note, since the cutoff of our inclusion criteria was at the end of 2022, we initially made reference to the Open Science Framework repository of 1 paper [82]; however, during review in 2023, this paper was published and we decided to link to the published version instead. Furthermore, our research is constrained to the literature published in English. Second, while our search strategy encompassed prominent databases such as PubMed, Scopus, and Web of Science and included additional material with a focus on policy documents, there may be relevant literature not included in our search, including gray literature, news articles, and blog posts. It must also be recognized that the assessment and categorization of papers, as well as the identification of core thematic areas, involve an element of subjectivity. Despite rigorous methodology and intersubjective blinded coding, interpretational variations may exist. Moreover, it is worth noting that the review offers interpretations and recommendations for considering and applying ethics within the field of infodemic management solely based on the ethical considerations and approaches identified within the analyzed body of literature. Given the rapid evolution of this field, it is essential to acknowledge that many pertinent aspects related to the ethics of infodemic management have not yet been thoroughly discussed. Therefore, we strongly encourage the research community, as well as infodemic managers and policy makers, to deepen our understanding of ethics within the context of infodemic management. This commitment to knowledge enhancement is essential for maintaining ethical standards and promoting responsible practices in an ever-changing information landscape. Furthermore, as previously discussed, most studies in our corpus originate from Western contexts, potentially limiting the generalizability of our findings to diverse global settings with distinct cultural and societal norms. Finally, published studies may not fully represent the spectrum of research or of the practice conducted in the field of infodemic management.

### Conclusions

Infodemic management presents relevant ethical challenges. The insights derived from our systematic scoping review highlight that ethical approaches in infodemic management and social listening are necessary for the medium- and long-term effectiveness of infodemic management practices. In this review, several fundamental and procedural ethical principles have been identified, including community engagement, education, inclusivity, equity, and trust, among others, all of which enhance the quality and efficacy of these crucial public health activities. Our review provides a foundational understanding of the ethical issues arising in infodemic management. It will hopefully contribute to improving ethical guidance in this field and help to adequately address these issues in future infodemic management programs. To fully realize the potential of ethical infodemic management, future research should strive for empirical studies and incorporate diverse global perspectives to further advance the field and protect public health during acute or chronic health events.

## Appendix

Multimedia Appendix 1 Queries, PRISMA (Preferred Reporting Items for Systematic Reviews and Meta-Analyses) checklist, supplementary figures, and other emerging themes.

Multimedia Appendix 2 Issues and aims.

## Abbreviations

EG: expert group
OSF: Open Science Framework
PRISMA: Preferred Reporting Items for Systematic Reviews and Meta-Analyses
WHO: World Health Organization

## Data Availability

All data are accessible within the manuscript, in the Open Science Framework repository, or in the accompanying multimedia appendix.
