# Supplementary material for: Ethical Considerations in Infodemic Management: Systematic Scoping Review
Source: JMIR Infodemiology. 2024 Aug 29;4:e56307. doi: 10.2196/56307 (PMC11393515; doi:10.2196/56307)
Supplement: Multimedia Appendix 1 [file infodemiology_v4i1e56307_app1.docx]

Ethical Considerations in Infodemic Management: A Systematic Scoping Review

Supplementary material

# Queries

## Query v1

"2002/01/01"[Date - Publication] : "2022/12/31"[Date - Publication]

**AND (**

("infodemic"[MeSH Terms] OR "infodemic"[All Fields] OR "infodemics"[All Fields] OR "infodemiology"[All Fields] OR "infoveillance"[All Fields])

**OR**

(("misinformation"[All Fields] OR "disinformation"[All Fields]) AND ("public health"[MeSH Terms] OR "information dissemination"[MeSH Terms] OR "knowledge management"[MeSH Terms] OR "communication"[MeSH Terms] OR "risk communication" [All Fields] OR "health information"[All Fields] OR "harm reduction"[All Fields] OR "crisis management"[All Fields] OR "risk perception" [All Fields] OR "prevention and control"[All Fields] OR "persuasion"[All Fields]) AND (("pandemic s"[All Fields] OR "pandemics"[MeSH Terms] OR "pandemics"[All Fields] OR "pandemic"[All Fields] OR "epidemic s"[All Fields] OR "epidemics"[MeSH Terms] OR "epidemics"[All Fields] OR "epidemic"[All Fields] OR "epidemiology"[MeSH Subheading] OR "epidemiology"[All Fields] OR "disease outbreaks"[MeSH Terms]) OR ("public health crisis"[All Fields] OR "infection control"[All Fields] OR "infection"[All Fields] OR "infectious diseases"[All Fields] OR "outbreak"[All Fields] OR "crisis"[All Fields] OR "risk"[All Fields] OR "emergency"[All Fields] OR "vulnerable populations"[All Fields] OR "safety"[All Fields] OR "uncertainty"[All Fields])))

**OR**

("social listening"[All Fields])

**)AND**

("ethic s"[All Fields] OR "ethical"[All Fields] OR "ethically"[All Fields] OR "ethics"[MeSH Terms] OR "ethics"[All Fields] OR "ethic"[All Fields] OR "ethics"[MeSH Subheading] OR "bioethics"[MeSH Terms] OR "public health ethics"[All Fields])

## Query v2

"2002/01/01"[Date - Publication] : "2022/12/31"[Date - Publication]

AND (

("infodemic"[MeSH Terms] OR "infodemic"[All Fields] OR "infodemics"[All Fields] OR "infodemiology"[All Fields] OR "infoveillance"[All Fields])

OR

(("misinformation"[All Fields] OR "disinformation"[All Fields] OR "information overload"[All Fields] OR "information pollution"[All Fields] OR "information quality"[All Fields] OR "information void"[All Fields] OR "information deficit"[All Fields]) AND (("information dissemination"[MeSH Terms] OR "knowledge management"[MeSH Terms] OR "communication"[MeSH Terms] OR "risk communication" [All Fields] OR "harm reduction"[All Fields] OR "crisis management"[All Fields] OR "risk perception" [All Fields] OR "prevention and control"[All Fields] OR "content analysis"[All Fields] OR "information verification"[All Fields] OR "public health response"[All Fields] OR "claim verification"[All Fields] OR "psychological support"[All Fields] OR "digital engagement"[All Fields] OR "cyber resilience"[All Fields] OR "consumer protection"[All Fields] OR "self efficacy"[All Fields] OR "self-efficacy"[All Fields] OR "knowledge translation"[All Fields] OR "science translation"[All Fields] OR "message amplification"[All Fields] OR "message dissemination"[All Fields] OR "message development"[All Fields] OR "fake news detection"[All Fields] OR "information science education"[All Fields] OR "information retrieval"[All Fields] OR "information literacy"[All Fields] OR "text analysis"[All Fields] OR "media literacy"[All Fields] OR "knowledge engineering"[All Fields] OR "topic modelling"[All Fields] OR "bot detection"[All Fields] OR "context analysis"[All Fields] OR "opinion mining"[All Fields] OR "predictive model"[All Fields] OR "cyber hygiene"[All Fields] OR "information hygiene"[All Fields] OR "misinformation detection"[All Fields]) OR ("social listening"[All Fields] OR "pandemic preparedness"[All Fields] OR "pandemic response"[All Fields] OR "social media listening"[All Fields] OR "social monitoring"[All Fields] OR "community engagement"[All Fields] OR "social media data analytics"[All Fields] OR "social media analysis"[All Fields] OR "social media research"[All Fields] OR ("public"[All Fields] AND ("sentiment"[All Fields] OR "sentiments"[All Fields]) OR "social media use"[All Fields] OR "social big data"[All Fields] OR "public perception"[All Fields] OR "social marketing" [All Fields] OR "public emotion"[All Fields] OR "public sentiment"[All Fields] OR "public concern"[All Fields] OR "public perception"[All Fields] OR "public opinion"[All Fields] OR "community emotion"[All Fields] OR "community sentiment"[All Fields] OR "community concern"[All Fields] OR "community question"[All Fields] OR "community opinion"[All Fields] OR "social sensing"[All Fields] OR "computational social science"[All Fields]))) AND ( ("pandemic s"[All Fields] OR "pandemics"[MeSH Terms] OR "pandemics"[All Fields] OR "pandemic"[All Fields] OR "epidemic s"[All Fields] OR "epidemics"[MeSH Terms] OR "epidemics"[All Fields] OR "epidemic"[All Fields] OR "epidemiology"[MeSH Subheading] OR "epidemiology"[All Fields] OR "disease outbreaks"[MeSH Terms] OR "infection control"[All Fields] OR "infection"[All Fields] OR "infectious diseases"[All Fields] OR "outbreak"[All Fields]) OR ("public health"[MeSH Terms] OR "public health crisis"[All Fields] OR "crisis"[All Fields] OR "risk"[All Fields] OR "emergency"[All Fields] OR "vulnerable populations"[All Fields] OR "safety"[All Fields] OR "uncertainty"[All Fields])))

)AND

("ethic s"[All Fields] OR "ethical"[All Fields] OR "ethically"[All Fields] OR "ethics"[MeSH Terms] OR "ethics"[All Fields] OR "ethic"[All Fields] OR "ethics"[MeSH Subheading] OR "bioethics"[MeSH Terms] OR "public health ethics"[All Fields])

## Query v3

"2002/01/01"[Date - Publication] : "2022/12/31"[Date - Publication]

**AND (**

("infodemic"[MeSH Terms] OR "infodemic"[All Fields] OR "infodemics"[All Fields] OR "infodemiology"[All Fields] OR "infoveillance"[All Fields])

**OR**

(("misinformation"[All Fields] OR "disinformation"[All Fields] OR "information overload"[All Fields] OR "information pollution"[All Fields] OR "information quality"[All Fields] OR "information void"[All Fields] OR "information deficit"[All Fields]) AND (("information dissemination"[MeSH Terms] OR "knowledge management"[MeSH Terms] OR "communication"[MeSH Terms] OR "risk communication" [All Fields] OR "harm reduction"[All Fields] OR "crisis management"[All Fields] OR "risk perception" [All Fields] OR "prevention and control"[All Fields] OR "content analysis"[All Fields] OR "information verification"[All Fields] OR "public health response"[All Fields] OR "claim verification"[All Fields] OR "psychological support"[All Fields] OR "digital engagement"[All Fields] OR "cyber resilience"[All Fields] OR "consumer protection"[All Fields] OR "self efficacy"[All Fields] OR "self-efficacy"[All Fields] OR "knowledge translation"[All Fields] OR "science translation"[All Fields] OR "message amplification"[All Fields] OR "message dissemination"[All Fields] OR "message development"[All Fields] OR "fake news detection"[All Fields] OR "information science education"[All Fields] OR "information retrieval"[All Fields] OR "information literacy"[All Fields] OR "text analysis"[All Fields] OR "media literacy"[All Fields] OR "knowledge engineering"[All Fields] OR "topic modelling"[All Fields] OR "bot detection"[All Fields] OR "context analysis"[All Fields] OR "opinion mining"[All Fields] OR "predictive model"[All Fields] OR "cyber hygiene"[All Fields] OR "information hygiene"[All Fields] OR "misinformation detection"[All Fields] OR "digital disease surveillance"[All Fields] OR "netnography"[All Fields] OR "information manipulation"[All Fields] OR "digital influence"[All Fields]) OR ("social listening"[All Fields] OR "pandemic preparedness"[All Fields] OR "pandemic response"[All Fields] OR "social media listening"[All Fields] OR "social monitoring"[All Fields] OR "community engagement"[All Fields] OR "social media data analytics"[All Fields] OR "social media analysis"[All Fields] OR "social media research"[All Fields] OR ("public"[All Fields] AND ("sentiment"[All Fields] OR "sentiments"[All Fields]) OR "social media use"[All Fields] OR "social big data"[All Fields] OR "public perception"[All Fields] OR "social marketing" [All Fields] OR "public emotion"[All Fields] OR "public sentiment"[All Fields] OR "public concern"[All Fields] OR "public perception"[All Fields] OR "public opinion"[All Fields] OR "community emotion"[All Fields] OR "community sentiment"[All Fields] OR "community concern"[All Fields] OR "community question"[All Fields] OR "community opinion"[All Fields] OR "social sensing"[All Fields] OR "computational social science"[All Fields] OR "social media mining"[All Fields] OR "social media analytics"[All Fields] OR "social networking services"[All Fields] OR "online social networking"[All Fields] OR "social network technology"[All Fields] OR "social audit"[All Fields]))) AND ( ("pandemic s"[All Fields] OR "pandemics"[MeSH Terms] OR "pandemics"[All Fields] OR "pandemic"[All Fields] OR "epidemic s"[All Fields] OR "epidemics"[MeSH Terms] OR "epidemics"[All Fields] OR "epidemic"[All Fields] OR "epidemiology"[MeSH Subheading] OR "epidemiology"[All Fields] OR "disease outbreaks"[MeSH Terms] OR "infection control"[All Fields] OR "infection"[All Fields] OR "infectious diseases"[All Fields] OR "outbreak"[All Fields]) OR ("public health"[MeSH Terms] OR "public health crisis"[All Fields] OR "crisis"[All Fields] OR "risk"[All Fields] OR "emergency"[All Fields] OR "vulnerable populations"[All Fields] OR "safety"[All Fields] OR "uncertainty"[All Fields] OR "public health communication"[All Fields]))))

**)AND**

("ethic s"[All Fields] OR "ethical"[All Fields] OR "ethically"[All Fields] OR "ethics"[MeSH Terms] OR "ethics"[All Fields] OR "ethic"[All Fields] OR "ethics"[MeSH Subheading] OR "bioethics"[MeSH Terms] OR "public health ethics"[All Fields] OR "moral"[All Fields] OR "morally"[All Fields] OR "morality"[All Fields] OR "democracy"[All Fields] OR "GDPR infringement"[All Fields] OR "unlawful data disclosure"[All Fields] OR "malicious data disclosure"[All Fields] OR "identity protection"[All Fields] OR "privacy"[All Fields] OR "justice"[All Fields] OR "fairness"[All Fields])

## Translated queries

### Web of Science

TS=((("infodemic" OR "infodemics" OR "infodemiology" OR "infoveillance") OR (("misinformation" OR "disinformation" OR "information overload" OR "information pollution" OR "information quality" OR "information void" OR "information deficit") AND ("information dissemination" OR "knowledge management" OR "communication" OR "risk communication" OR "harm reduction" OR "crisis management" OR "risk perception" OR "prevention and control" OR "content analysis" OR "information verification" OR "public health response" OR "claim verification" OR "psychological support" OR "digital engagement" OR "cyber resilience" OR "consumer protection" OR "self efficacy" OR "self-efficacy" OR "knowledge translation" OR "science translation" OR "message amplification" OR "message dissemination" OR "message development" OR "fake news detection" OR "information science education" OR "information retrieval" OR "information literacy" OR "text analysis" OR "media literacy" OR "knowledge engineering" OR "topic modelling" OR "bot detection" OR "context analysis" OR "opinion mining" OR "predictive model" OR "cyber hygiene" OR "information hygiene" OR "misinformation detection" OR "digital disease surveillance" OR "netnography" OR "information manipulation" OR "digital influence")) OR (("social listening" OR "pandemic preparedness" OR "pandemic response" OR "social media listening" OR "social monitoring" OR "community engagement" OR "social media data analytics" OR "social media analysis" OR "social media research" OR ("public" AND ("sentiment" OR "sentiments")) OR "social media use" OR "social big data" OR "public perception" OR "social marketing" OR "public emotion" OR "public sentiment" OR "public concern" OR "public perception" OR "public opinion" OR "community emotion" OR "community sentiment" OR "community concern" OR "community question" OR "community opinion" OR "social sensing" OR "computational social science" OR "social media mining" OR "social media analytics" OR "social networking services" OR "online social networking" OR "social network technology" OR "social audit"))) AND (("pandemic s" OR "pandemics" OR "pandemic" OR "epidemic s" OR "epidemics" OR "epidemic" OR "epidemiology" OR "disease outbreaks" OR "infection control" OR "infection" OR "infectious diseases" OR "outbreak") OR ("public health" OR "public health crisis" OR "crisis" OR "risk" OR "emergency" OR "vulnerable populations" OR "safety" OR "uncertainty" OR "public health communication")) AND ("ethic s" OR "ethical" OR "ethically" OR "ethics" OR "ethic" OR "bioethics" OR "public health ethics" OR "moral" OR "morally" OR "morality" OR "democracy" OR "GDPR infringement" OR "unlawful data disclosure" OR "malicious data disclosure" OR "identity protection" OR "privacy" OR "justice" OR "fairness")) AND PY=(2002-2022) AND MC=(Human Medicine Medical Sciences OR Philosophy And Ethics OR Allied Medical Sciences OR Infection OR Public Health OR Government And Law OR Medical Sciences OR Epidemiology OR Human Medicine OR Behavior OR Information Studies OR Sociology OR Computer Applications OR Education OR Pollution Assessment Control And Management OR Models And Simulations OR Climatology OR Anthropology OR Hospital Administration OR Human Ecology OR Communication OR Ecology Environmental Sciences OR Sanitation OR General Life Studies OR Linguistics OR Terrestrial Ecology OR Waste Management)

### Scopus

TITLE-ABS-KEY((("infodemic" OR "infodemics" OR "infodemiology" OR "infoveillance") OR (("misinformation" OR "disinformation" OR "information overload" OR "information pollution" OR "information quality" OR "information void" OR "information deficit") AND ("information dissemination" OR "knowledge management" OR "communication" OR "risk communication" OR "harm reduction" OR "crisis management" OR "risk perception" OR "prevention and control" OR "content analysis" OR "information verification" OR "public health response" OR "claim verification" OR "psychological support" OR "digital engagement" OR "cyber resilience" OR "consumer protection" OR "self efficacy" OR "self-efficacy" OR "knowledge translation" OR "science translation" OR "message amplification" OR "message dissemination" OR "message development" OR "fake news detection" OR "information science education" OR "information retrieval" OR "information literacy" OR "text analysis" OR "media literacy" OR "knowledge engineering" OR "topic modelling" OR "bot detection" OR "context analysis" OR "opinion mining" OR "predictive model" OR "cyber hygiene" OR "information hygiene" OR "misinformation detection" OR "digital disease surveillance" OR "netnography" OR "information manipulation" OR "digital influence")) OR (("social listening" OR "pandemic preparedness" OR "pandemic response" OR "social media listening" OR "social monitoring" OR "community engagement" OR "social media data analytics" OR "social media analysis" OR "social media research" OR ("public" AND ("sentiment" OR "sentiments")) OR "social media use" OR "social big data" OR "public perception" OR "social marketing" OR "public emotion" OR "public sentiment" OR "public concern" OR "public perception" OR "public opinion" OR "community emotion" OR "community sentiment" OR "community concern" OR "community question" OR "community opinion" OR "social sensing" OR "computational social science" OR "social media mining" OR "social media analytics" OR "social networking services" OR "online social networking" OR "social network technology" OR "social audit"))) AND (("pandemic s" OR "pandemics" OR "pandemic" OR "epidemic s" OR "epidemics" OR "epidemic" OR "epidemiology" OR "disease outbreaks" OR "infection control" OR "infection" OR "infectious diseases" OR "outbreak") OR ("public health" OR "public health crisis" OR "crisis" OR "risk" OR "emergency" OR "vulnerable populations" OR "safety" OR "uncertainty" OR "public health communication")) AND ("ethic s" OR "ethical" OR "ethically" OR "ethics" OR "ethic" OR "bioethics" OR "public health ethics" OR "moral" OR "morally" OR "morality" OR "democracy" OR "GDPR infringement" OR "unlawful data disclosure" OR "malicious data disclosure" OR "identity protection" OR "privacy" OR "justice" OR "fairness")) AND PUBYEAR > 2002 PUBYEAR < 2022 AND SUBJAREA (medi OR heal OR comp OR deci OR psyc OR soci) AND (LIMIT-TO(DOCTYPE,"ar"))

# PRISMA checklist

| **Section and Topic** | **Item #** | **Checklist item** | **Location where item is reported** |
| --- | --- | --- | --- |
| **TITLE** | | |  |
| Title | 1 | Identify the report as a systematic review. | Title page |
| **ABSTRACT** | | |  |
| Abstract | 2 | See the PRISMA 2020 for Abstracts checklist. | Abstract (only relevant information included) |
| **INTRODUCTION** | | |  |
| Rationale | 3 | Describe the rationale for the review in the context of existing knowledge. | Introduction |
| Objectives | 4 | Provide an explicit statement of the objective(s) or question(s) the review addresses. | Introduction |
| **METHODS** | | |  |
| Eligibility criteria | 5 | Specify the inclusion and exclusion criteria for the review and how studies were grouped for the syntheses. | Methods / data retrieval and screening of records; Methods / screening of full texts |
| Information sources | 6 | Specify all databases, registers, websites, organisations, reference lists and other sources searched or consulted to identify studies. Specify the date when each source was last searched or consulted. | Methods / query definition; Methods / query translation |
| Search strategy | 7 | Present the full search strategies for all databases, registers and websites, including any filters and limits used. | Methods / query definition; Methods / query translation; Supplementary material |
| Selection process | 8 | Specify the methods used to decide whether a study met the inclusion criteria of the review, including how many reviewers screened each record and each report retrieved, whether they worked independently, and if applicable, details of automation tools used in the process. | Methods / data retrieval and screening of records; Methods / screening of full texts |
| Data collection process | 9 | Specify the methods used to collect data from reports, including how many reviewers collected data from each report, whether they worked independently, any processes for obtaining or confirming data from study investigators, and if applicable, details of automation tools used in the process. | Methods / paper assessment; Methods / analysis; Supplementary material |
| Data items | 10a | List and define all outcomes for which data were sought. Specify whether all results that were compatible with each outcome domain in each study were sought (e.g. for all measures, time points, analyses), and if not, the methods used to decide which results to collect. | Methods / paper assessment |
|  | 10b | List and define all other variables for which data were sought (e.g. participant and intervention characteristics, funding sources). Describe any assumptions made about any missing or unclear information. | Methods / paper assessment |
| Study risk of bias assessment | 11 | Specify the methods used to assess risk of bias in the included studies, including details of the tool(s) used, how many reviewers assessed each study and whether they worked independently, and if applicable, details of automation tools used in the process. | Discussion / Limitations |
| Effect measures | 12 | Specify for each outcome the effect measure(s) (e.g. risk ratio, mean difference) used in the synthesis or presentation of results. | NA |
| Synthesis methods | 13a | Describe the processes used to decide which studies were eligible for each synthesis (e.g. tabulating the study intervention characteristics and comparing against the planned groups for each synthesis (item #5)). | Methods / paper assessment; Methods / analysis |
|  | 13b | Describe any methods required to prepare the data for presentation or synthesis, such as handling of missing summary statistics, or data conversions. | Methods / paper assessment |
|  | 13c | Describe any methods used to tabulate or visually display results of individual studies and syntheses. | Methods / paper assessment |
|  | 13d | Describe any methods used to synthesize results and provide a rationale for the choice(s). If meta-analysis was performed, describe the model(s), method(s) to identify the presence and extent of statistical heterogeneity, and software package(s) used. | NA |
|  | 13e | Describe any methods used to explore possible causes of heterogeneity among study results (e.g. subgroup analysis, meta-regression). | NA |
|  | 13f | Describe any sensitivity analyses conducted to assess robustness of the synthesized results. | NA |
| Reporting bias assessment | 14 | Describe any methods used to assess risk of bias due to missing results in a synthesis (arising from reporting biases). | NA |
| Certainty assessment | 15 | Describe any methods used to assess certainty (or confidence) in the body of evidence for an outcome. | NA |
| **RESULTS** | | |  |
| Study selection | 16a | Describe the results of the search and selection process, from the number of records identified in the search to the number of studies included in the review, ideally using a flow diagram. | Results / characterization of included papers |
|  | 16b | Cite studies that might appear to meet the inclusion criteria, but which were excluded, and explain why they were excluded. | Results / characterization of included papers |
| Study characteristics | 17 | Cite each included study and present its characteristics. | Figures and tables |
| Risk of bias in studies | 18 | Present assessments of risk of bias for each included study. | Discussion / limitations |
| Results of individual studies | 19 | For all outcomes, present, for each study: (a) summary statistics for each group (where appropriate) and (b) an effect estimate and its precision (e.g. confidence/credible interval), ideally using structured tables or plots. | NA |
| Results of syntheses | 20a | For each synthesis, briefly summarise the characteristics and risk of bias among contributing studies. | NA |
|  | 20b | Present results of all statistical syntheses conducted. If meta-analysis was done, present for each the summary estimate and its precision (e.g. confidence/credible interval) and measures of statistical heterogeneity. If comparing groups, describe the direction of the effect. | NA |
|  | 20c | Present results of all investigations of possible causes of heterogeneity among study results. | NA |
|  | 20d | Present results of all sensitivity analyses conducted to assess the robustness of the synthesized results. | NA |
| Reporting biases | 21 | Present assessments of risk of bias due to missing results (arising from reporting biases) for each synthesis assessed. | Discussion; Discussion / limitations |
| Certainty of evidence | 22 | Present assessments of certainty (or confidence) in the body of evidence for each outcome assessed. | NA |
| **DISCUSSION** | | |  |
| Discussion | 23a | Provide a general interpretation of the results in the context of other evidence. | Discussion |
|  | 23b | Discuss any limitations of the evidence included in the review. | Discussion / limitations |
|  | 23c | Discuss any limitations of the review processes used. | Discussion / limitations |
|  | 23d | Discuss implications of the results for practice, policy, and future research. | Discussion |
| **OTHER INFORMATION** | | |  |
| Registration and protocol | 24a | Provide registration information for the review, including register name and registration number, or state that the review was not registered. | The review was not registered; however, we kept detailed research logs on an OSF repository: <https://osf.io/28d73/> |
|  | 24b | Indicate where the review protocol can be accessed, or state that a protocol was not prepared. | The protocol is available on the study’s OSF repository: <https://osf.io/28d73/> |
|  | 24c | Describe and explain any amendments to information provided at registration or in the protocol. | Methods / query definition; Methods / query translation |
| Support | 25 | Describe sources of financial or non-financial support for the review, and the role of the funders or sponsors in the review. | Acknowledgements |
| Competing interests | 26 | Declare any competing interests of review authors. | Conflict of Interest |
| Availability of data, code and other materials | 27 | Report which of the following are publicly available and where they can be found: template data collection forms; data extracted from included studies; data used for all analyses; analytic code; any other materials used in the review. | Methods; supplementary materials |

*From:*  Page MJ, McKenzie JE, Bossuyt PM, Boutron I, Hoffmann TC, Mulrow CD, et al. The PRISMA 2020 statement: an updated guideline for reporting systematic reviews. BMJ 2021;372:n71. doi: 10.1136/bmj.n71

# Figure S1


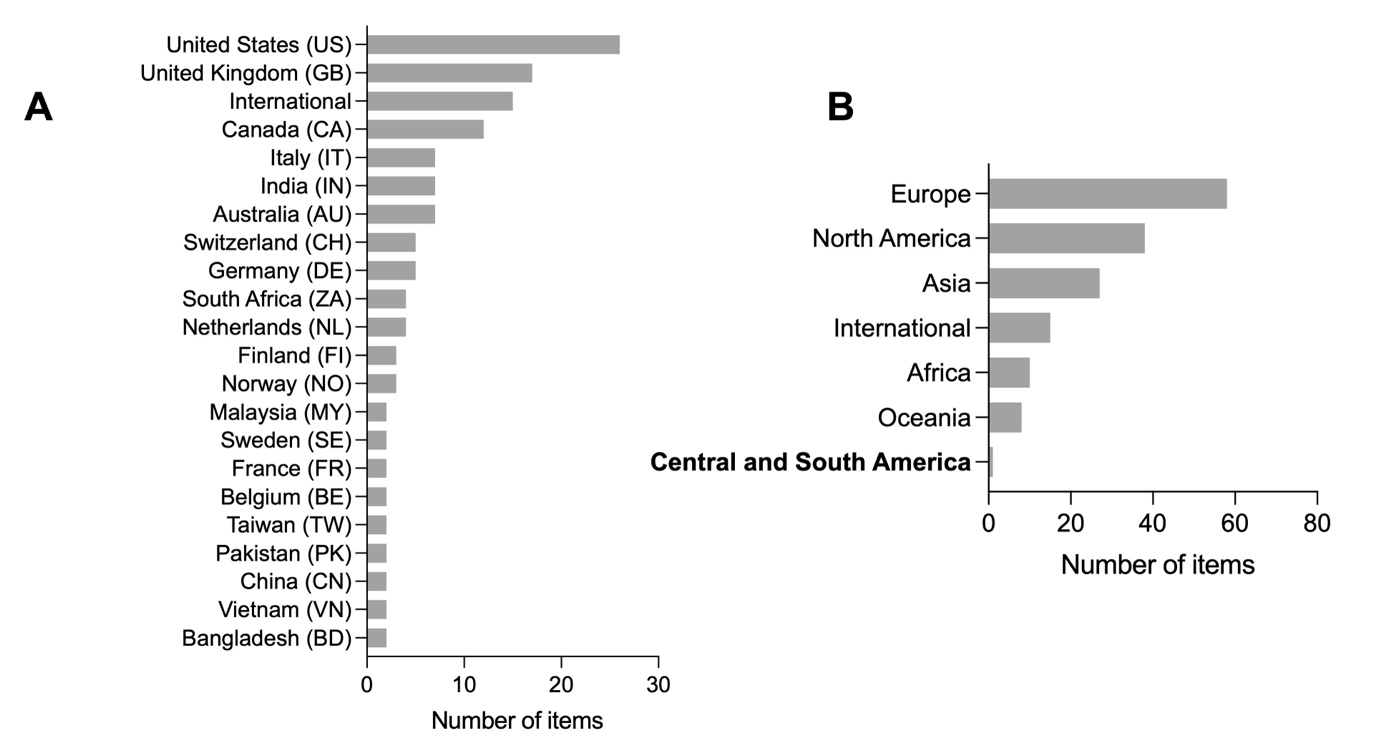


**Figure S1. Geographical origin of the papers included in the review.** The United States leads the ranking, followed by the United Kingdom and by international organizations (A). Breaking down the analysis for continents, Europe is the most prolific one, followed by North America and Asia. Central and South America has the lowest output of papers on ethics in infodemic management and social listening.

# Other emerging themes

In the following sections, we discuss the ethical principles and themes highlighted in Cluster 2 (**Figure 3B**), which is the group of the second most represented issues in the body of literature analyzed in our systematic scoping review.

## Community engagement

The literature identifies four core goals of community engagement: enhancing citizens' protection, improving individual health outcomes, fostering trust and legitimacy, and sharing responsibility. These goals guide the development of community engagement strategies and ensure that they align with the needs and expectations of the community.

Foremost, the literature emphasizes the importance of rejecting a top-down paternalistic approach in favor of inclusive, bottom-up strategies when providing information to the targeted publics. The preference for such approaches embodies the principle of respecting the autonomy and voice of the community [1–3]. This paradigm shift in information dissemination underscores the need to inform information strategies through community insights rather than dictating them from above, without prior knowledge of how they will be received by the target publics, and whether the intended outcome reflects the expectations of the initiator of the message [4].
In this context, given the dynamic nature of community engagement, the literature advocates for continued research to identify and enhance efficient methods of community and public engagement. The absence of clearly defined, universally applicable methods underscores the need for continuous innovation and adaptation. Existing approaches to community engagement encompass a spectrum of methods, including surveys, qualitative methods (e.g., focus groups and interviews), and deliberative forums, often complemented by social media engagement. Surveys and qualitative methods have proven instrumental in shaping policy decision-making by capturing public opinion and sentiment. Deliberative forums, while valuable, serve as a countermeasure to address the concern of public misunderstanding of complex issues. The literature highlights that by providing participants with detailed information prior to soliciting their opinions, deliberative forums contribute to a more informed and nuanced public perspective [5]. The literature underscores the value of adopting multiple methods of public engagement in a complementary manner, ensuring a comprehensive understanding of a community's views [6]. Two-way communication between public health entities and communities emerges as a central element of ethical community engagement [7]. This communication involves not only disseminating information but also actively seeking community input. The values and concerns of the community must be considered at every stage of planning, implementation, and data usage from surveillance and social listening [1].
In addition, collaboration between health departments, healthcare providers, and community groups that prioritize local perspectives is pivotal. These collaborations facilitate the tailoring of health messaging and offer insights into how these messages resonate within the community [8–10].
Furthermore, the promotion of community and individual engagement with research practices and evidence-based arguments is underscored. Encouraging communities to actively participate in discussions surrounding public health and infodemic management contributes to more informed and well-rounded decision-making [7,10,11].

Besides, an ethical approach to community engagement places a significant emphasis on the inclusion of vulnerable and marginalized groups in the design of engagement efforts. This not only empowers these often underserved populations but also enhances the overall representativeness of the engagement process [8,10].

*Active* public participation, as facilitated by active social listening practices, is generally recommended by the literature over *passive* forms of engagement [3,5,6]. Active engagement underscores the importance of listening, understanding, and responding to the concerns of the community, by directly engaging with the community, rather than extrapolating data from, e.g. social media users, without them being aware of it.

Ultimately, community engagement is portrayed as an ongoing process of relationship building that is indispensable at every operational step. It ensures that public health measures are not only grounded in ethics and community needs, but also more effective.

## Empowerment through education

Although education is not inherently an ethical concept, its enhancement is strongly linked to fostering individual autonomy, thereby empowering individuals to make ethically sound decisions in the face of complex information challenges. A fundamental challenge identified in the literature pertains to the absence of substantial evidence regarding the skills required to foster information literacy. Furthermore, there is a lack of clarity surrounding the most effective education plans, strategies, and designs for building these skills [12]. The literature highlights the need to develop and promote robust research frameworks aimed at resolving these open questions and addressing these uncertainties. By doing so, educational efforts can be grounded in evidence and best practices, ensuring their effectiveness in the long term.
Education is seen as a potent tool in the fight against misinformation. The literature emphasizes the importance of teaching and training both the public and medical professionals in information literacy and digital literacy [13–15]. Equipping the public with the necessary skills to find reliable information, identify misinformation, and assess scientific evidence is paramount [16]. Medical professionals, in particular, play an important role in guiding patients towards these goals [17,18]. The overarching aim is to reduce the influence of fake news and misinformation on governments, industries, and private actors.First of all, although education is not inherently an ethical concept, its enhancement is strongly linked to fostering individual autonomy, thereby empowering individuals to make ethically sound decisions in the face of complex information challenges. A fundamental challenge identified in the literature pertains to the absence of substantial evidence regarding the skills required to foster information literacy. Furthermore, there is a lack of clarity surrounding the most effective education plans, strategies, and designs for building these skills [12]. The literature highlights the need to develop and promote robust research frameworks aimed at resolving these open questions and addressing these uncertainties. By doing so, educational efforts can be grounded in evidence and best practices, ensuring their effectiveness in the long term.
Education is seen as a potent tool in the fight against misinformation. The literature emphasizes the importance of teaching and training both the public and medical professionals in information literacy and digital literacy [13–15]. Equipping the public with the necessary skills to find reliable information, identify misinformation, and assess scientific evidence is paramount [16]. Medical professionals, in particular, play an important role in guiding patients towards these goals [17,18]. The overarching aim is to reduce the influence of fake news and misinformation on governments, industries, and private actors.

As mentioned, promoting and teaching critical thinking skills emerges as a central pillar of education in the context of infodemic management. This form of education and training is vital in building resilience against misinformation [14]. For some papers in the corpus of the literature, it encompasses capacity building, pre-bunking, fostering self-efficacy in adhering to recommended health behaviors, and the ability to develop risk perception [19–21]. Therefore, critical thinking skills empower individuals to question, evaluate, and discern the validity of information they encounter, promoting a more discerning and informed public.

In essence, education is important to build a resilient and well-informed community. It provides the knowledge and skills necessary for individuals to navigate the complex information landscape, critically evaluate the information they encounter, and make informed decisions. By fostering information literacy, improving communication effectiveness, and nurturing critical thinking, educational initiatives play a relevant role in promoting ethical, effective, and sustainable infodemic management practices [22]. Importantly, integrating educational strategies as a primary goal in infodemic management, and improving the information ecosystem, would have a positive effect on societal resilience against misinformation and fake news, thus permitting public health institutions and governments to reduce the need for short-lived solutions to decrease exposure to dangerous health information [4]. Avoiding solutions that are effective in the short-term but ineffective and detrimental in the long-term, allows infodemic management practices to be grounded in a more trustworthy and healthier information ecosystem, contributing to reduce societal polarization.

## Transparency

Transparency plays an important role in the ethical landscape of infodemic management. First of all, the literature highlights the necessity of clarifying the purpose of the objectives behind infodemic management and social listening practices; these need to clearly defined, to safeguard public trust [23,23–25]. The principle of transparency is key in the context of privacy and data collection, underlining the necessity of making citizens fully aware of how their data is acquired, stored, and employed, enabling them to make informed decisions about data sharing [24,26,27]. Even when data is collected from social media platforms, data transparency remains a continual requirement, ensuring clarity about data sources, handling, and dissemination to uphold the integrity of data-driven decisions [26,28].

Furthermore, the literature highlights how the transparency of communication strategies is crucial; for example, national communication strategies should be openly and publicly accessible, ensuring public scrutiny to the government's approach in managing the infodemic [4,29,30].

Another emerged aspect of transparency involves the disclosure of conflicts of interest, obliging organizations and decision-makers to openly declare any affiliations or interests that might influence their actions or decisions, fostering trust and integrity [10,31,32].

Importantly, work in the reviewed corpus of literature highlights that transparency is a universally acknowledged principle, transcending cultural differences [9]. In essence, transparency is the ethical foundation that upholds the integrity of information, fosters public trust, and promotes ethical decision-making within the realm of infodemic management.

## Privacy

The theme of privacy within the sphere of infodemic management and in the context of social listening brings to light a series of ethical considerations and pragmatic strategies essential in navigating the intricate balance between data collection and public trust. The literature highlights critical findings and measures, each instrumental in safeguarding the privacy rights of individuals while maintaining the effectiveness of data-driven approaches.
A primary ethical consideration is the need to strike a delicate balance between data retention and tracking on one hand, and the preservation of privacy on the other. This balance encompasses a fundamental choice between a "data-first" and "privacy-first" approach [33]. Finding this equilibrium is essential to ensure that data collection practices are both effective and respectful of individual privacy rights.
To fortify privacy protections, the literature underscores the importance of ensuring that citizens are not only aware of their privacy rights but also understand how data is collected and utilized [34–36]. This transparency-based approach serves as a foundational element in promoting informed and ethical data practices, allowing individuals to make informed choices about their own data.

Confidentiality and anonymity are crucial when gathering data through active and passive social listening. Active social listening practices often involve interactions with individuals, making it even more imperative to safeguard the confidentiality of the data collected. Additionally, to ensure the privacy and anonymity of individuals, steps must be taken to guarantee non-reidentification, further preventing the misuse or exposure of sensitive information [6].

In most cases, the literature encourages the obtaining of informed consent as an ethical practice [6,26,30]. Rather than viewing informed consent as an obstacle to the effectiveness of social listening practices, it should be considered an aim in infodemic management. By ensuring that individuals are informed and consent to data collection, the erosion of institutional trust is mitigated [34,36]. This practice not only respects the privacy rights of individuals but also reinforces public trust in data-driven approaches.

In essence, the principle of privacy underscores the importance of protecting the privacy rights of individuals while harnessing the power of data-driven practices in infodemic management. The literature suggests that striking a balance between data and privacy, promoting awareness of privacy rights, ensuring confidentiality and anonymity, and obtaining informed consent are relevant measures in preserving the ethical integrity of data collection practices.

## Free speech versus regulation

Key findings and principles underscore the ethical considerations surrounding the preservation of free speech, regulation, and public trust.
The fundamental principle of allowing a pluralism of voices and opinions to emerge in the public debate stands as a cornerstone of free speech in the literature [37–39]. Embracing diverse perspectives is vital for fostering a robust and democratic discourse. The literature underscores that this diversity is essential in the context of infodemic management, as it also allows for a comprehensive understanding of public concerns and viewpoints [37–39]. Censorship, content moderation, deplatforming, and banning, while they may be employed with good intentions, are identified by most of the papers in the literature that addressed the issue, as practices that can erode public trust [4,21,40]. The heavy-handed use of these measures, which can be beneficial during the acute phase of a crisis in which the public has no to little resilience against misinformation [39,41,42], in the medium- and long-term can lead to perceptions of bias and undermine the public's confidence in the transparency and fairness of information platforms, leading to a reduction of trust for those institutions that aim to safeguard individual and public health, thus potentially undermining the effectiveness of their future efforts [4,21]. In light of this, the literature advocates for a cautious approach to content removal and user banning, even in the face of misinformation. This stems from the recognition that stringent measures may infringe upon the principle of freedom of expression. Furthermore, such practices can create difficulties for social listening efforts aimed at identifying population concerns. There is a risk, which has occurred during the pandemic, that individuals and groups who feel stifled may migrate to alternative communication platforms with fewer regulations, ultimately fragmenting the information landscape and leading to further societal polarization [4,6,43]. Balancing the preservation of free speech with the necessity to curb the spread of misinformation is a nuanced endeavor [21]. Encouraging open dialogue and discourse, together with promoting educational strategies to improve public resilience against misinformation, fosters a culture of critical thinking and self-regulation, where individuals are better equipped to discern credible information from misinformation [22,39].
Key findings and principles underscore the ethical considerations surrounding the preservation of free speech, regulation, and public trust.
The fundamental principle of allowing a pluralism of voices and opinions to emerge in the public debate stands as a cornerstone of free speech in the literature [37–39]. Embracing diverse perspectives is vital for fostering a robust and democratic discourse. The literature underscores that this diversity is essential in the context of infodemic managementIM, as it also allows for a comprehensive understanding of public concerns and viewpoints [37–39]. Censorship, content moderation, deplatforming, and banning, while they may be employed with good intentions, are identified by most of the papers in the literature that addressed the issue, as practices that can erode public trust [4,21,40]. The heavy-handed use of these measures, which can be beneficial during the acute phase of a crisis in which the public has no to little resilience against misinformation [39,41,42], in the medium- and long-term can lead to perceptions of bias and undermine the public's confidence in the transparency and fairness of information platforms, leading to a reduction of trust for those institutions that aim to safeguard individual and public health, thus potentially undermining the effectiveness of their future efforts [4,21]. In light of this, the literature advocates for a cautious approach to content removal and user banning, even in the face of misinformation. This stems from the recognition that stringent measures may infringe upon the principle of freedom of expression. Furthermore, such practices can create difficulties for social listeningSL efforts aimed at identifying population concerns. There is a risk, which has occurred during the pandemic, that individuals and groups who feel stifled may migrate to alternative communication platforms with fewer regulations, ultimately fragmenting the information landscape and leading to further societal polarization [4,6,43]. Balancing the preservation of free speech with the necessity to curb the spread of misinformation is a nuanced endeavor [21]. Encouraging open dialogue and discourse, together with promoting educational strategies to improve public resilience against misinformation, fosters a culture of critical thinking and self-regulation, where individuals are better equipped to discern credible information from misinformation [22,39].
In summary, the theme of free speech versus regulation underscores the need for a balanced and cautious approach. While protecting the principle of free speech and the diversity of voices is essential, it is equally important to address the challenges posed by misinformation. This calls for a measured and ethical approach that preserves public trust and democratic discourse while mitigating the harm caused by the infodemic.

## Informing versus manipulating behavior

Respecting the autonomy of individuals is identified as a fundamental ethical procedural principle. This principle necessitates the protection of individuals' ability to make independent, informed decisions without manipulation. Thus, fostering information literacy and the enhancement of critical thinking skills are pivotal in enabling individuals to navigate the complexities of the information landscape. Empowered with these skills, individuals can discern credible information from misinformation, thereby preserving their autonomy without the need of being manipulated into a desirable (and positive) health behavior [36,38,44,45].
Simultaneously, while respecting individual autonomy, some of the literature recognizes the necessity to influence individual knowledge, attitudes, beliefs, and behaviors to increase compliance with evidence-based public health measures. This influence aims to support informed choices that safeguard individual and community health. This includes the promotion of healthy lifestyles, which, when communicated ethically, contributes to the greater good. This strategy is particularly important during acute health crises with a public that is little to no resilient against misinformation [32,38,46].
However, in this sense a critical aspect highlighted in the literature is the importance of credibility and trust. As information providers, institutions must strive to enhance their credibility and trustworthiness. The effectiveness of communication strategies depends significantly on the level of trust in the source [32]. In contexts of high trust, communication attempts are perceived as providing information for the greater good. In contrast, in contexts of low trust, the same communication may be perceived as manipulation tactics. Hence, the ethical responsibility to cultivate trust is central to the successful dissemination of information while preserving autonomy [36,46,47].Respecting the autonomy of individuals is identified as a fundamental ethical procedural principle. This principle necessitates the protection of individuals' ability to make independent, informed decisions without manipulation. Thus, fostering information literacy and the enhancement of critical thinking skills are pivotal in enabling individuals to navigate the complexities of the information landscape. Empowered with these skills, individuals can discern credible information from misinformation, thereby preserving their autonomy without the need of being manipulated into a desirable (and positive) health behavior [36,38,44,45].
Simultaneously, while respecting individual autonomy, some of the literature recognizes the necessity to influence individual knowledge, attitudes, beliefs, and behaviors to increase compliance with evidence-based public health measures. This influence aims to support informed choices that safeguard individual and community health. This includes the promotion of healthy lifestyles, which, when communicated ethically, contributes to the greater good. This strategy is particularly important during acute health crises with a public that is little to no resilient against misinformation [32,38,46].
However, in this sense a critical aspect highlighted in the literature is the importance of credibility and trust. As the information provider, institutions must strive to enhance their credibility and trustworthiness. The effectiveness of communication strategies depends significantly on the level of trust in the source [32]. In contexts of high trust, communication attempts are perceived as providing information for the greater good. In contrast, in contexts of low trust, the same communication may be perceived as manipulation tactics. Hence, the ethical responsibility to cultivate trust is central to the successful dissemination of information while preserving autonomy [36,46,47].

In conclusion, in an optimal scenario, the delicate balance between informing and influencing behavior requires the ethical preservation of individual autonomy, coupled with the responsible dissemination of information to promote informed choices [4,44]. Information literacy and trust-building are essential underpinnings, ensuring that the public is empowered to navigate the information ecosystem while also making informed decisions that safeguard individual and community health.

## Honesty and conflicts of interest

Honesty is at the core of ethical infodemic management. The literature underscores the critical importance of ensuring that no one within an organization harbors the intent to deceive or lie to those served by the organization. The consequences of reputational damage, even when caused by individuals within a large organization, can have a profound and detrimental effect on public trust. This principle is particularly salient in the context of risk and crisis communication, where the integrity of information must be guaranteed. Honesty is the bedrock upon which public trust is constructed, and the violation of this principle can have far-reaching consequences for the effectiveness of infodemic management [32,48].
The ultimate goal of every organization involved in infodemic management must be to safeguard public health. This overarching objective serves to guide decision-making to ensure that public health takes precedence over any other interests.
Another aspect of honesty involves refraining from intentionally causing harm to a political or geopolitical opponent. Deliberate actions aimed at damaging opponents inevitably result in societal polarization and the erosion of trust in a specific segment of the population [49]. Thus, ethical conduct entails focusing on public health goals rather than pursuing political or geopolitical goals.

Conflicts of interest are therefore identified as significant ethical pitfalls that can undermine public trust. These conflicts can manifest in various forms, including involvement in politics or the presence of decision-making processes biased on political grounds. The literature underscores that operating with conflicts of interest not only erodes institutional trust but may also compromise the integrity and impartiality of information and decision-making processes.

## Good governance

For good governance in the context of infodemic management, the literature highlights the significance of ethical governance practices to ensure transparency, accountability, and the integrity of information dissemination.
A theme emerged from the analysis of the literature is that ethical governance upholds democratic principles, ensuring that decision-making processes and information dissemination are inclusive and transparent [11,20,26,50]. It calls for engaging stakeholders, respecting diverse perspectives, and promoting open and participatory approaches to decision-making [51]. By adhering to democratic principles, governance structures foster public trust and collective responsibility in navigating the complexities of the infodemic [51].

As a second theme, ethical governance of data curation and dissemination emerged as essential in the digital age. This involves responsible collection, management, and sharing of data while protecting privacy and ensuring data security. Good ethical governance requires measures to prevent data breaches and unauthorized access, preserving individual rights. Furthermore, it entails verifying the accuracy and reliability of data to maintain the public's trust in information sources. To achieve this, transparency in data collection methods, responsible data storage, and the ethical use of data for decision-making and communication is needed [20,26,50].

A key aspect that emerged from the literature review concerning good governance is the relevance of ensuring the truthfulness of contents provided through information campaigns. This involves robust fact-checking, source verification, and content review processes to ensure that information disseminated to the public is accurate and free from misinformation [26,39,48,51]. Ethical governance practices also encompass addressing inaccuracies promptly and transparently, thereby upholding the integrity of information. This control of truthfulness is a critical element in preserving public trust in information sources and decision-makers.

In summary, good governance in infodemic management revolves around democratizing access to information, responsible data management, and the promotion of truthfulness in content. These principles collectively contribute to transparency, accountability, and the integrity of information, fostering public trust and responsible decision-making.

# Bibliography

1. Charania NA, Tsuji LJ. A community-based participatory approach and engagement process creates culturally appropriate and community informed pandemic plans after the 2009 H1N1 influenza pandemic: Remote and isolated First Nations communities of sub-arctic Ontario, Canada. BMC Public Health. 2012. PMID:22472012

2. Cohen CB. The ethics of human reproductive cloning: When world views collide. Account Res. Taylor and Francis Inc.; 2004. p. 183–199. PMID:15812964

3. Dupras C, Williams-Jones B. The Expert and the Lay Public: Reflections on Influenza A (H1N1) and the Risk Society. Am J PUBLIC Health 2012 Apr;102(4):591–595. doi: 10.2105/AJPH.2011.300417

4. Spitale G, Germani F, Biller-Andorno N. The PHERCC Matrix. An Ethical Framework for Planning, Governing, and Evaluating Risk and Crisis Communication in the Context of Public Health Emergencies. Am J Bioeth 2023;1–16. doi: https://doi.org/10.1080/15265161.2023.2201191

5. Abboah-Offei M, Gyasi Darkwa A, Ayim A, Ansah-Ofei AM, Dovlo D, Awoonor-Williams JK, Agongo EEA, Agyepong IA, Elsey H. Adapting the Community-based Health Planning and Services (CHPS) to engage poor urban communities in Ghana: Protocol for a participatory action research study. BMJ Open. BMJ Publishing Group; 2021. PMID:34315798

6. Spitale G, Biller-Andorno N, Germani F. Concerns Around Opposition to the Green Pass in Italy: Social Listening Analysis by Using a Mixed Methods Approach. J Med Internet Res Canada; 2022 Feb 16;24(2):e34385. PMID:35156930

7. Spitale G, Merten S, Jafflin K, Schwind B, Kaiser-Grolimund A, Biller-Andorno N. A Novel Risk and Crisis Communication Platform to Bridge the Gap Between Policy Makers and the Public in the Context of the COVID-19 Crisis (PubliCo): Protocol for a Mixed Methods Study. JMIR Res Protoc Canada; 2021 Nov 1;10(11):e33653. PMID:34612823

8. Dash S, Parray AA, De Freitas L, Mithu MIH, Rahman MM, Ramasamy A, Pandya AK. Combating the COVID-19 infodemic: A three-level approach for low and middle-income countries. BMJ Glob Health. BMJ Publishing Group; 2021. doi: 10.1136/bmjgh-2020-004671

9. Lor A, Thomas JC, Barrett DH, Ortmann LW, Herrera Guibert DJ. Key ethical issues discussed at CDC-sponsored international, regional meetings to explore cultural perspectives and contexts on pandemic influenza preparedness and response. Int J Health Policy Manag. Kerman University of Medical Sciences; 2016. p. 653–662. PMID:27801360

10. Vanderslott S, Van Ryneveld M, Marchant M, Lees S, Nolna SK, Marsh V. How can community engagement in health research be strengthened for infectious disease outbreaks in Sub-Saharan Africa? A scoping review of the literature. BMC Public Health. BioMed Central Ltd; 2021. PMID:33794820

11. WHO. An ad hoc WHO technical consultation managing the COVID-19 infodemic: call for action. 2020. Available from: https://www.who.int/publications-detail-redirect/9789240010314 [accessed Jan 30, 2023]

12. Willingham DT. Ask the Cognitive Scientist: How Can Educators Teach Critical Thinking? Am Fed Teach 2023 Jul 12; Available from: https://www.aft.org/ae/fall2020/willingham [accessed Nov 20, 2023]

13. Ali S, Khalid A, Zahid E. Is COVID-19 Immune to Misinformation? A Brief Overview. Asian Bioeth Rev Springer; 2021;13(2):255–277. doi: 10.1007/s41649-020-00155-x

14. Germani F, Biller-Andorno N. How to counter the anti-vaccine rhetoric: Filling information voids and building resilience. Hum Vaccines Immunother United States; 2022 Nov 30;18(6):2095825. PMID:35802046

15. Wilhelm E, Ballalai I, Belanger M-E, Benjamin P, Bertrand-Ferrandis C, Bezbaruah S, Briand S, Brooks I, Bruns R, Bucci LM, Calleja N, Chiou H, Devaria A, Dini L, D’Souza H, Dunn AG, Eichstaedt JC, Evers SMAA, Gobat N, Gissler M, Gonzales IC, Gruzd A, Hess S, Ishizumi A, John O, Joshi A, Kaluza B, Khamis N, Kosinska M, Kulkarni S, Lingri D, Ludolph R, Mackey T, Mandić-Rajčević S, Menczer F, Mudaliar V, Murthy S, Nazakat S, Nguyen T, Nilsen J, Pallari E, Taschner NP, Petelos E, Prinstein MJ, Roozenbeek J, Schneider A, Srinivasan V, Stevanović A, Strahwald B, Abdul SS, Machiri SV, Linden S van der, Voegeli C, Wardle C, Wegwarth O, White BK, Willie E, Yau B, Purnat TD. Measuring the Burden of Infodemics: Summary of the Methods and Results of the Fifth WHO Infodemic Management Conference. JMIR Infodemiology 2023 Feb 20;3(1):e44207. doi: 10.2196/44207

16. Spitale G, Germani F, Biller-Andorno N. Can AI Disinform Us Better? OSF; 2022 Oct 19; doi: 10.17605/OSF.IO/HV6ZY

17. Green J, Petty J, Whiting L, Orr F, Smart L, Brown A-M, Jones L. “Blurred boundaries”: When nurses and midwives give anti-vaccination advice on Facebook. Nurs Ethics England; 2022 May;29(3):552–568. PMID:35142239

18. Calabrese SK, Mayer KH, Marcus JL. Prioritising pleasure and correcting misinformation in the era of U=U. Lancet HIV Netherlands; 2021 Mar;8(3):e175–e180. PMID:33662266

19. Cohen ERM, Masum H, Berndtson K, Saunders V, Hadfield T, Panjwani D, Persad DL, Minhas GS, Daar AS, Singh JA, Singer PA. Public engagement on global health challenges. BMC Public Health. 2008. PMID:18492256

20. WHO. WHO competency framework: Building a response workforce to manage infodemics. 2021. Available from: https://www.who.int/publications-detail-redirect/9789240035287 [accessed Jan 30, 2023]

21. WHO. WHO public health research agenda for managing infodemics. 2021. Available from: https://www.who.int/publications-detail-redirect/9789240019508 [accessed Jan 30, 2023]

22. World Health Organization. Regional Office for Europe. Digital solutions to health risks raised by the COVID-19 infodemic: policy brief. World Health Organization. Regional Office for Europe; 2022. Report No.: WHO/EURO:2022-5351-45116-64364. Available from: https://apps.who.int/iris/handle/10665/356315 [accessed Jan 30, 2023]

23. UN informal interagency dialogues on disinformation. Shared UN considerations for online communications companies on the issues of countering disinformation and enhancing transparency. 2021. Available from: https://www.un.org/techenvoy/sites/www.un.org.techenvoy/files/general/UN_InteragencyDialogue2_v2.pdf

24. UN Interagency Dialogue on Disinformation and Data Transparency. Selection of data from online platforms that would enable better understanding of disinformation online and efforts to counter it. 2021. Available from: https://www.un.org/techenvoy/sites/www.un.org.techenvoy/files/general/UN_InteragencyDialogue1_v2_0.pdf

25. Knudsen J, Perlman -Gabel Maddie, Uccelli IG, Jeavons J, Chokshi DA. Combating Misinformation as a Core Function of Public Health. NEJM Catal Massachusetts Medical Society; 2023;4(2):CAT.22.0198. doi: 10.1056/CAT.22.0198

26. WHO. WHO guidelines on ethical issues in public health surveillance. WHO; 2017. Available from: https://www.who.int/publications/i/item/who-guidelines-on-ethical-issues-in-public-health-surveillance

27. Ummer O, Scott K, Mohan D, Chakraborty A, Lefevre AE. Connecting the dots: Kerala’s use of digital technology during the COVID-19 response. BMJ Glob Health. BMJ Publishing Group; 2021. doi: 10.1136/bmjgh-2021-005355

28. ITU. Digital tools and strategies in COVID-19 infodemic response: Case studies and discussion. 2021. Available from: https://www.itu.int:443/en/publications/ITU-D/Pages/publications.aspx [accessed Jan 30, 2023]

29. Taylor L. The price of certainty: How the politics of pandemic data demand an ethics of care. Big Data Soc. SAGE Publications Ltd; 2020. doi: 10.1177/2053951720942539

30. Taylor J, Pagliari C. Mining social media data: How are research sponsors and researchers addressing the ethical challenges? Res Ethics 2018 Apr;14(2):1–39. doi: 10.1177/1747016117738559

31. Sambala EZ, Manderson L. Ethical Problems in Planning for and Responses to Pandemic Influenza in Ghana and Malawi. Ethics Behav. Routledge; 2018. p. 199–217. doi: 10.1080/10508422.2016.1274993

32. Oxman AD, Fretheim A, Lewin S, Flottorp S, Glenton C, Helleve A, Vestrheim DF, Iversen BG, Rosenbaum SE. Health communication in and out of public health emergencies: to persuade or to inform? Health Res Policy Syst England; 2022 Mar 5;20(1):28. PMID:35248064

33. Fahey RA, Hino A. COVID-19, digital privacy, and the social limits on data-focused public health responses. Int J Inf Manag. Elsevier Ltd; 2020. doi: 10.1016/j.ijinfomgt.2020.102181

34. Grande D, Mitra N, Marti XL, Merchant R, Asch D, Dolan A, Sharma M, Cannuscio C. Consumer Views on Using Digital Data for COVID-19 Control in the United States. JAMA Netw Open. American Medical Association; 2021. PMID:34009347

35. Health Enabled. Finding the signal through the noise. A landscape review and framework to enhance the effective use of digital social listening for immunisation demand generation. 2021. Available from: https://www.who.int/news/item/18-08-2021-social-listening-finding-the-signal-through-the-noise

36. Hendl T, Chung R, Wild V. Pandemic Surveillance and Racialized Subpopulations: Mitigating Vulnerabilities in COVID-19 Apps. J Bioethical Inq. Springer; 2020. p. 829–834. PMID:32840858

37. Purnat TD, Nguyen T, Briand S, editors. Managing Infodemics in the 21st Century: Addressing New Public Health Challenges in the Information Ecosystem. Cham: Springer International Publishing; 2023. doi: 10.1007/978-3-031-27789-4ISBN:978-3-031-27788-7

38. Lor P, Wiles B, Britz J. Re-thinking Information Ethics: Truth, conspiracy theories, and librarians in the Covid-19 era. Libri. De Gruyter Saur; 2021. p. 1–14. doi: 10.1515/libri-2020-0158

39. Mills MC, Sivelä J. Should spreading anti-vaccine misinformation be criminalised? The BMJ. BMJ Publishing Group; 2021. PMID:33597153

40. Niemiec E. COVID-19 and misinformation: Is censorship of social media a remedy to the spread of medical misinformation? EMBO Rep England; 2020 Nov 5;21(11):e51420. PMID:33103289

41. Stevens G, O’Donnell VL, Williams L. Public domain or private data? Developing an ethical approach to social media research in an inter-disciplinary project. Educ Res Eval. Routledge; 2015. p. 154–167. doi: 10.1080/13803611.2015.1024010

42. Stommel W, Rijk L de. Ethical approval: none sought. How discourse analysts report ethical issues around publicly available online data. Res Ethics SAGE Publications Ltd; 2021 Jul 1;17(3):275–297. doi: 10.1177/1747016120988767

43. Monti C, Cinelli M, Valensise C, Quattrociocchi W, Starnini M. Online conspiracy communities are more resilient to deplatforming. PNAS Nexus 2023 Oct 1;2(10):pgad324. doi: 10.1093/pnasnexus/pgad324

44. Biller-Andorno N, Spitale G. Addressing Volatile Ethical Issues of Covid-19 with the Core Five Enduring Values List for Health Care Professionals. NEJM Catal Innov Care Deliv Massachusetts Medical Society; 2022 Aug 4; Available from: https://catalyst.nejm.org/doi/full/10.1056/CAT.22.0108 [accessed Aug 5, 2022]

45. Marco-Franco JE, Pita-Barros P, Vivas-Orts D, González-de-Julián S, Vivas-Consuelo D. COVID-19, Fake News, and Vaccines: Should Regulation Be Implemented? Int J Environ Res Public Health Switzerland; 2021 Jan 16;18(2). PMID:33467179

46. Morley J, Cowls J, Taddeo M, Floridi L. Public Health in the Information Age: Recognizing the Infosphere as a Social Determinant of Health. J Med Internet Res Canada; 2020 Aug 3;22(8):e19311. PMID:32648850

47. Purnat T, Nguyen T, Ishizumi A, Yau B, White B, Cecchini S, Samuel R, Hess S, Bezbaruah S, Briand S. Delivering actionable infodemic insights and recommendations for the COVID-19 pandemic response. 2022. Available from: https://reliefweb.int/report/world/weekly-epidemiological-record-wer-8-july-2022-vol-97-no-27-pp-313-324-enfr [accessed Jan 30, 2023]

48. Lovari A, Bowen SA. Social media in disaster communication: A case study of strategies, barriers, and ethical implications. J Public Aff. John Wiley and Sons Ltd; 2020. doi: 10.1002/pa.1967

49. Sniečkutė M, Gaižauskaitė I. Covid-19 Crisis: Government’s (Dis)Trust In The People And Pitfalls of Liberal Democracies. Partecip E Conflitto. University of Salento; 2021. p. 152–175. doi: 10.1285/i20356609v14i1p152

50. Curkovic M, Kosec A, Roje Bedekovic M, Bedekovic V. Epistemic responsibilities in the COVID-19 pandemic: Is a digital infosphere a friend or a foe? J Biomed Inform United States; 2021 Mar;115:103709. PMID:33571677

51. Posada A, Lopez Inigo R, Sport J. Turning Social Listening Data into Action: Barriers and Recommendations Observed through a COVID-19 Rumor Response - World | ReliefWeb. 2023. Available from: https://reliefweb.int/report/world/turning-social-listening-data-action-barriers-and-recommendations-observed-through-covid-19-rumor-response [accessed Mar 17, 2023]
